# Intermediate Detection in the Casiopeina–Cysteine Interaction Ending in the Disulfide Bond Formation and Copper Reduction

**DOI:** 10.3390/molecules26195729

**Published:** 2021-09-22

**Authors:** Lillian G. Ramírez-Palma, Adrián Espinoza-Guillén, Fabiola Nieto-Camacho, Alexis E. López-Guerra, Virginia Gómez-Vidales, Fernando Cortés-Guzmán, Lena Ruiz-Azuara

**Affiliations:** 1 Instituto de Química, Universidad Nacional Autónoma de México, Av. Universidad 3000, C. U., México City 04510, Mexico; lila.gis.rp@gmail.com (L.G.R.-P.); gomvidal@unam.mx (V.G.-V.); 2 Centro Conjunto de Investigación en Química Sustentable UAEMex-UNAM, Carretera Toluca-Atlacomulco km 14.5, Toluca 50200, Mexico; 3 Facultad de Química, Universidad Nacional Autónoma de México, Av. Universidad 3000, C. U., México City 04510, Mexico; adrianeg24@gmail.com (A.E.-G.); fabille.nc@gmail.com (F.N.-C.); eduga_9@live.com.mx (A.E.L.-G.)

**Keywords:** copper complexes, glutathione depletion, cysteine, Casiopeina, intermediate, copper reduction

## Abstract

A strategy to improve the cancer therapies involves agents that cause the depletion of the endogenous antioxidant glutathione (GSH), increasing its efflux out of cells and inducing apoptosis in tumoral cells due to the presence of reactive oxygen species. It has been shown that Casiopeina copper complexes caused a dramatic intracellular GSH drop, forming disulfide bonds and reducing Cu^II^ to Cu^I^. Herein, through the determination of the [Cu^II^]–SH bond before reduction, we present evidence of the adduct between cysteine and one Casiopeina as an intermediate in the cystine formation and as a model to understand the anticancer activity of copper complexes. Evidence of such an intermediate has never been presented before.

## 1. Introduction

**Figure 1:**
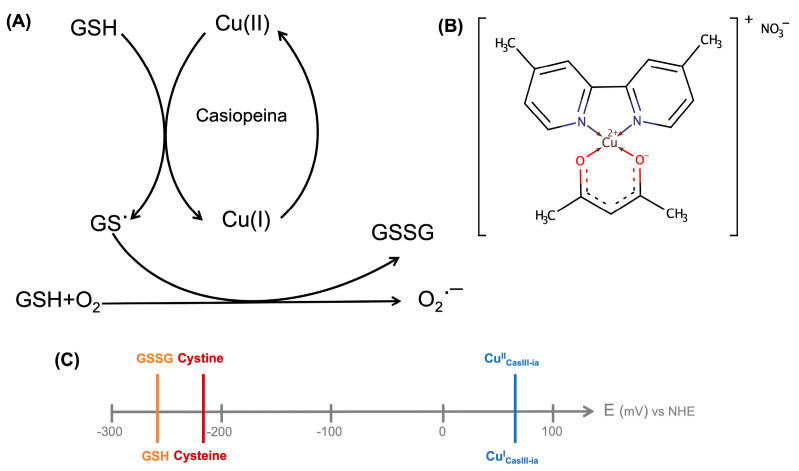
(**A**) The hypothesis of the interaction between the Casiopeina family and a thiolated redactor [6]. (**B**) CasIII-ia structure. (**C**) The redox potential of thiol-containing biomolecules and CasIII-ia.

The study of the oxidation of endogenous thiolated compounds, such as glutathione (GSH) and cysteine (Cys), is essential to understand the oxidative stress within the cells [1]. Glutathione (γ-l-glutamyl-l-cysteinyl-glycine, GSH) is a tripeptide that participates in redox processes into the cells, where the Cys residues of GSH are easily oxidized to disulfide (GSSG) [2,3]. It also participates in cancer cell protection against xenobiotics, ionizing radiations, and oxidative stress. Its oxidation favors the opening of the mitochondrial permeability transition pore complex, facilitating the release of death-related molecular signals [4,5]. A strategy to improve cancer therapies’ efficacy should involve cytosolic and mitochondrial GSH depletion through an increase of GSH efflux out of cells [4]. Kachadourian and coworkers tested, in human lung cancer cells (H157 and A549), one copper complex of the Casiopeina^®^ family, showing that it induced a dramatic drop in the intracellular levels of GSH (Figure 1A) [6]. Additionally, there are reports of GSH depletion produced by copper complexes on cervix HeLa and neuroblastoma CHP-212. GSSG/GSH and cystine/cysteine’s redox potentials are very similar, −263 and −220 mV vs. normal hydrogen electrode (NHE) respectively [7,8], with glutathione being a slightly better reducing agent (Figure 1C). The oxidation by copper(II) ions of cysteine-containing peptides such as glutathione and cysteine has been studied to understand this biometal’s role in oxidative stress processes [9,10,11,12]. The copper favors the oxidation of thiols [11,13], where Cu^II^ reacts with thiols to form [Cu^II^]–thiol adducts depending on their molar ratio. The [Cu^II^]–thiol complex is reduced to Cu^I^, and in turn, thiols are oxidized to the corresponding radicals. The CasIII-ia ([Cu(4,4′-dimethyl-2,2′-bipyridine)(acetylacetonate)]NO_3_*(H_2_O)_2_, Figure 1B) is a copper(II) complex from the Casiopeina family with a potential of 62 mV vs. NHE [14], which could mediate the oxidation of biological thiols (Figure 1C). Recently, a paper [15] demonstrated the formation of O_2_**·**^−^ when bisdiimine copper(II) chelates were reduced by ascorbate. However, the pathway by which Cu^II^ is reduced to Cu^I^ has not yet been described; therefore, in this work, we present a strategy to understand this reduction process and determine the possible intermediate.

These observations suggest the importance of copper complexes, such as the Casiopeinas, to oxidate thiol residues, inducing mitochondrial damage [6]. From the above considerations, in the present work, we study the specific interaction between cysteine and CasIII-ia to understand the pathway leading to a final reduction of the copper metal center and the disulfide bond formation. We focused on Cys, the reactive site of GSH, because it reacts slower than GSH in solution, and standard analytic techniques can detect its reactive intermediates. Additionally, we used mechanochemical methods to reduce the oxidation reaction rate. The family of Casiopeínas^®^ [16] was designed considering three elements: copper(II) as a central metal and two mixed ligands with several substituents, providing to copper the possibility of producing cytotoxicity through different mechanisms of action [17]. Ligands modify the cationic copper’s transport properties [18], the electronic properties of the central metal [19], and the molecular recognition of the complex [20]. The Casiopeina activity goes along with the cytotoxic effects, such as the generation of reactive oxygen species (ROS) [21,22], which can damage cellular components through oxidation and alter the oxidation-reduction balance cell or interfere with the mechanisms of cellular signaling related to the redox state [23]. There are reports about the antiproliferative and antineoplastic activities over murine and xenografted human tumors [24,25]. On the other hand, survival was evaluated in murine models: L1210 (leukemia), S180 (sarcoma), and B16 (melanoma) [26]. In non-tumor cells, the mean inhibitory concentration of this compound was 4.7, determined in lymphocytes, approximately 250 times higher than that observed in tumor lines, suggesting a selectivity towards tumoral cells [27]. Additionally, pharmacokinetic analyses performed with rat urine [28] and dog blood [29] samples have proved a high elimination rate of the Casiopeinas. All these results indicate an increase in the in vivo activity of the compound CasIII-ia, concerning the cisplatin activity as a positive control, evaluated in the same system. Our group has investigated the relationship between the features of the metal complex and their activity. A QSAR study showed that the half-wave potential and aromatic ring in the molecule are relevant for the compounds’ action [14].

Other derived models can predict mixed chelate copper complexes’ degree of activity based on the chemical correlation between structure, EPR, and electrochemical behavior, supported by DFT calculations [19]. We also developed a regression model to reproduce the antiproliferative activity involving the atomic delocalization and dipole moment changes within the ligands’ C-N bonds [30]. These bonds are also determinant for the recognition site of copper complexes by the DNA backbone [20].

## 2. Results and Discussion

In solution, the endogenous reductant species, such as GSH, react very fast with Casiopeinas to be detected by standard analytical methods. For this reason, we decided to focus on the reactive site of GSH and Cys, and perform the reaction process in the solid state to observe the steps occurring in the Cys oxidation process. It has been reported that the Cys redox reaction barrier is smaller than the GSH one and also that the redox process is faster than any ligand exchange. In this way, Cys is an acceptable model of GSH for the process catalyzed by the CasIII-ia.

The CasIII-ia was prepared by the patent procedure [31,32,33]. To prove the inclusion of Cys in the copper coordination sphere and determine the features of the [Cu^II^]–Cys interaction, we performed powder X-ray diffraction (PXRD), electronic spectra (UV-Vis-NIR), infrared spectroscopy (FTIR), electron paramagnetic resonance spectroscopy (EPR), and mass spectra-direct analysis in real-time (MS-DART) analyses. To understand the structural evolution of the [Cu^II^]–Cys adduct, we carried out a DFT computational analysis for the cooper reduction process.

**Figure 2:**
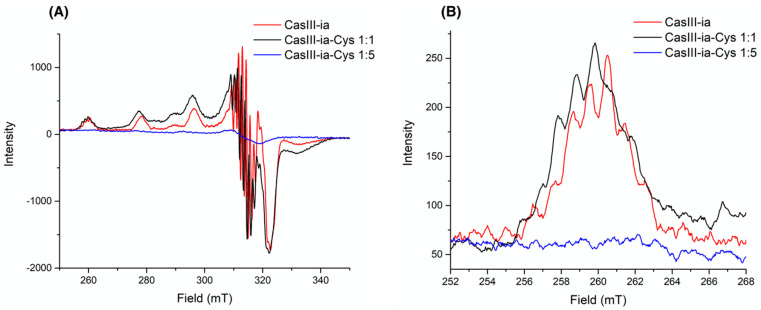
Experimental EPR spectra for CasIII-ia (1 mM) in red, CasIII-ia–Cys (1:1) in black, and CasIII-ia–Cys (1:5) in blue, in MeOH/H_2_O (1:1) frozen solution at 77 K. (**A**) From 250 to 350 mT, and (**B**) from 252 to 268 mT.

**Figure 3:**
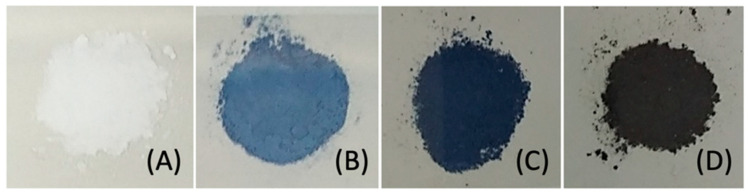
Reactant and intermediates of the mechanochemical solid-state reaction process: (**A**) cysteine (white), (**B**) CasIII-ia (Pantone 2139c), (**C**) intermediate 1 (Pantone 289c), and (**D**) intermediate 2 (Pantone 532c).

The reaction between equimolar solutions of Cys (colorless) and CasIII-ia (blue) showed color changes from brown/green to blue color (see the Movie S1 in the Supplementary Materials), and the same observations were reported by Seko et al. [34] and Ugone et al. [35]. EPR spectra of fresh mixtures of two CasIII-ia and Cys stoichiometric solutions were recorded at 77 K. Figure 2 shows the copper electronic environment change of CasIII-ia when it interacts with Cys. Therefore, it is proposed that CasIII-ia generates a [Cu^II^]–Cys adduct before its reduction. However, the reaction’s rapid kinetics avoids studying the [Cu^II^]–Cys adduct in solution using this technique. Then, we opted to change the conditions to reduce the reaction rate with a mechanochemical solid-state approach. CasIII-ia and Cys were milled until a homogenous solid mixture was obtained, EtOH was added, and was mixed until dryness. During the mechanical process, it was possible to observe a color change, from Pantone 2139c to Pantone 289c (Figure 3), that can be associated with Cys’ coordination to CasIII-ia. It was impossible to isolate and purify the reaction intermediates; therefore, we used several techniques to identify them within the reaction mixture.

First, we analyzed the powder X-Ray diffraction patterns of the reactants and the reaction mixture. The crystal square pyramid structure of CasIII-ia has been reported in a previous description (CCDC 1440021). Supplementary Figure S1 shows the diffraction pattern of the reaction mixture and the two reactants. It is possible to observe that the crystalline arrangement is conserved. Some remnant signals can be associated with the Cys and the CasIII-ia; however, some signals are no longer present, such as the 7.9 intense signals in 2θ. On the other hand, new signals are presented, such as 11.24, 12.12, 17.18, 25.94, 32.58, 33.02, 37.92, and 38.58 (see Supplementary Materials for details). These peaks confirm a new species that still have copper(II) as a metal center.

**Figure 4:**
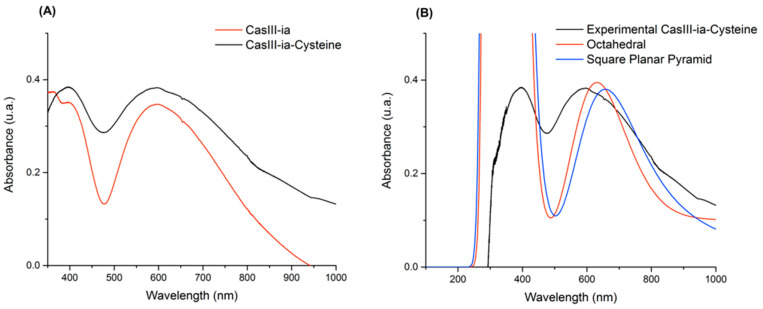
(**A**) UV-Vis spectra for CasIII-ia (red) and the reaction between CasIII-ia and cysteine (black). (**B**) UV-Vis spectra comparison between experimental reaction (black) and calculated geometries for the CasIII-ia–Cysteine system: octahedral (red) and square planar pyramid (blue).

The second evidence of the presence of a new copper(II) complex is the UV-Vis spectrum of the reaction mixture, as shown in Figure 4. The reaction mixture spectrum resembles that for CasIII-ia–Cys, with maxima at 395 and 598 nm. The latter can be associated with the electronic transitions of an elongated D_4h_ octahedral structure. The simulated spectra show that the signals of two possible CasIII-ia–Cys adduct arrangements, octahedral (axial Cys) and square planar pyramid (equatorial Cys), are very close to the experimental ones, 626 and 663 nm, respectively. The deconvolution of the two observed signals agrees with three theoretical excited states of octahedral and square planar pyramid geometries. These signals are mainly related to the transition to SOMO or LUMO molecular orbitals (for octahedral geometry: SOMO-20 → SOMO, SOMO-16 → SOMO, and SOMO-16 → LUMO; for square planar pyramid: SOMO-18 → SOMO, SOMO-18 → LUMO, and SOMO-15 → LUMO). Details are provided in the Supplementary Materials.

**Figure 5:**
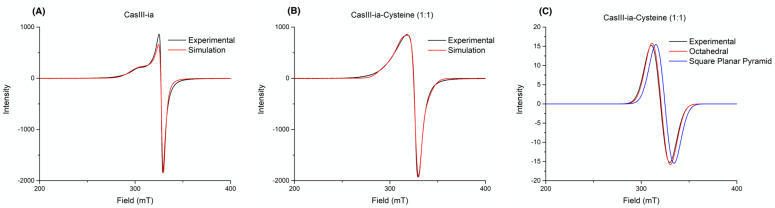
EPR spectra in the solid-state for (**A**) CasIII-ia and (**B**) CasIII-ia with cysteine reaction, and (**C**) comparison between experimental reaction and calculated geometries for the CasIII-ia–Cysteine system.

The third evidence of the interaction between CasIII-ia and Cys is the EPR data. Figure 5 shows the experimental spectra and fitting models of CasIII-ia (A) and CasIII-ia–Cys (B). Table 1 presents experimental and computational values for the g and A tensors of the spectra in Figure 5. We compared the observed spectra with DFT simulations of octahedral and square planar pyramid structures. CasIII-ia shows an axial symmetry spectrum associated with a square planar pyramid geometry. The reaction product is a 1:1 CasIII-ia/CasIII-ia–Cys mixture in the solid state. The linear combination of the axial and isotropic EPR profiles of CasIII-ia and CasIII-ia–Cys reproduce the experimental spectrum. The axial symmetry can be associated with geometry with a pseudo-Janh-Teller effect, where a Cys occupies the axial position of CasIII-ia. The difference between the experimental and theoretical g values ranges from 0.004 to 0.10, and for A values, from 25 to 98 MHz. These differences agree with that obtained in other reports [36,37]. For the CasIII-ia–Cys, we compared the experimental data with two different geometries: octahedral and square planar pyramid. The difference between the experimental and theoretical data is 0.004 and 0.030 for g_iso_, respectively. The A_iso_ value differences are 56 and 25 MHz, respectively. The best fit for g_iso_ corresponds to octahedral geometry, while for A_iso_ it is given by the square planar pyramid.

**Figure 6:**
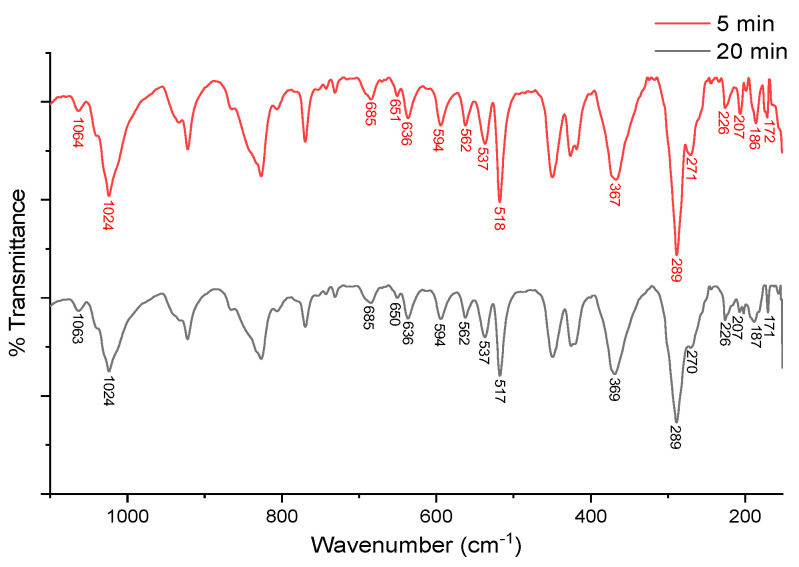
ATR-FTIR spectrum of CasIII-ia–Cys (1:1) mixture at 5 (red) and 20 (black) minutes.

The fourth evidence of the Cu–S interaction is the I.R. signals presented in the Supplementary Materials. Since cysteine is a molecule of biological interest, its solid-state vibrational spectra have been extensively studied, considering the polymorphisms that it can show [38] and the I.R. modifications with different protonation modes [39]. The Cys I.R. signals S-H (1063 cm^−1^), C-S (692 cm^−1^), and C-N (291 cm^−1^) [38], agreeing with theoretical frequencies (1056.73, 669.99, and 278 cm^−1^, respectively), yield the most significant changes when interacting with the copper complex. In the case of CasIII-ia, the Cu-O and C-N bonds’ I.R. signals appear at 596 and 294 cm^−1^ (603.88 and 279.73 cm^−1^ theoretical values), respectively. The 1:1 solid reaction mixture, with drops of EtOH, produces an adduct which presents I.R. signal variations as evidence of the CasIII-ia–Cys interaction (Figure 6). Based on computational information (179 and 194 cm^−1^ for octahedral and SPP theoretical values, respectively), we can assign the frequency at 187 cm^−1^ to the Cu–S interaction between CasIII-ia and Cys. It is possible to note signals associated with the metal–ligand interaction within CasIII-ia, such as Cu–O (594 cm^−1^) and Cu–N (289 cm^−1^). Cu–N weakened after Cys coordination from 294 to 289 cm^−1^. The weakening of the C-S bond is shown by reducing the frequency from 692 to 685 cm^−1^. One can observe a small S-H at 1063 cm^−1^ as evidence of unreacted Cys.

After NaOH was added in an equimolar amount, S-H frequency, at 1063 cm^−1^, disappeared, but the C-S and Cu-O vibrations remain. We confirmed that Cu–S interaction is associated with the 187 cm^−1^ frequency. These signals correlate with those exhibited by octahedral theoretical geometry. Details are provided in the Supplementary Materials.

**Figure 7:**
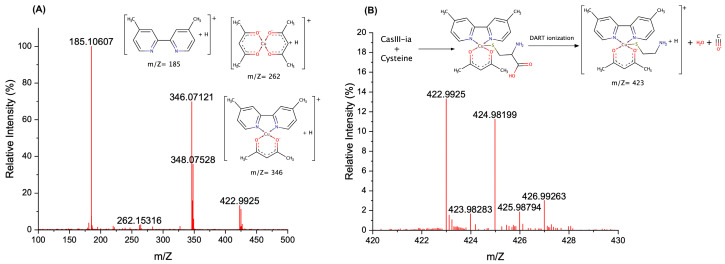
DART spectra of the mechanochemical solid-state mixture CasIII-ia–Cys at 200 °C. (**A**) From 100 to 500 m/Z, and (**B**) from 420 to 430 m/Z.

Our last evidence is from the direct analysis in real-time mass spectrometry (DART-MS). Under positive ionization of the DART technique, it has been reported that the amino-acid presents protonation, radical, and adduct formation [40]. In the Cys case, it is possible to observe [Cys + H]^+^ = 122 m/Z and [2Cys + H + H] = 243 m/Z. The mechanochemical solid-state mixture was analyzed using DART-MS, whose spectrum is presented in Figure 7. It is possible to observe the signal associated with dimethylbipyridine (m/Z = 185.1), Cu(acetylacetonate)_2_ (m/Z = 262), and CasIII-ia (m/Z = 346). At 200 °C, we found a signal of m/Z = 423 corresponding to the CasIII-ia–Cys adduct, with a loss of carboxylic group. It has been reported that Cys loses CO in situations where the sulfur atom is involved in a bond or a strong interaction. The fragmentation pattern of Cys by DART-MS and cystine by TANDEM-MS both present the [Cistina-H_2_O-CO + H] ion with m/Z = 195 when the CO loss can be observed [41,42]. The presence of the signals at 346 and 348 for CasIII-ia, at 262 and 264 for Cu(acetylacetonate)_2_, and at 422.99 and 424.98 for the adduct agree with the isotopic distribution for ^63^Cu and ^65^Cu observed in a copper(II) species. The abundance of each peak can be related to the stability of the analyzed species [43]. In this way, the adduct is an unstable species compared with the CasIII-ia.

**Figure 8:**
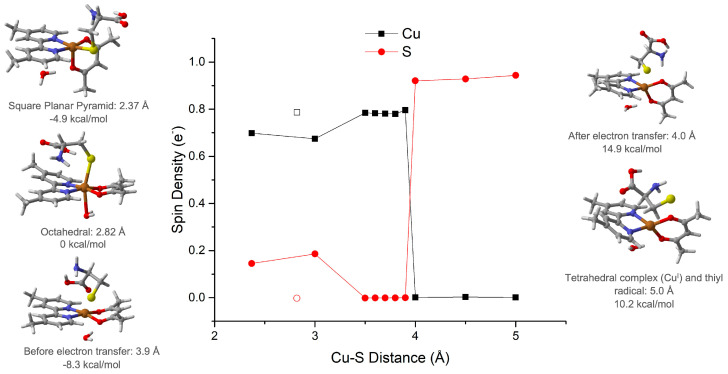
From square planar pyramid Cu^II^ complex to tetrahedral Cu^I^ complex, electron transfer path. Spin density values for copper atom in black and for sulfur atom in red.

To understand the pathway leading to a final reduction of the copper metal center, we calculated the structures and their dynamics. The two possible molecular arrangements of the CasIII-ia–Cys adduct are the octahedral and the square planar pyramid (Figure 8). In the former, the sulfur atom occupies an axial coordination position at 2.82 Å Cu–S distance. The latter presents the sulfur atom located in an equatorial position, at 2.37 Å Cu–S distance. The square planar pyramid is 4.9 kcal/mol more stable than octahedral geometry.

The reductive process’s reaction path begins from the Cys coordination to CasIII-ia in the square planar pyramid geometry, and it is presented in Figure 8, along with the atomic spin population. From the initial 2.37 Å, the Cu–S distance increases until 3.9 Å, where the electron is transferred from sulfur to the copper atom. It is possible to observe a spin population change from 0.78 to 0.001 e^−^ in the copper atom, which is transferred to the sulfur atom, which shows an increase from 0.00 to 0.94 e^−^. In this process, the copper valence shell changes, and then the geometry of the complex becomes tetrahedral. Our group has previously reported the spin change effect on the metal valence shell and thus on the complex structure [44,45]. From this point, the thiyl radical is free to participate in the following reaction to form the disulfide bond. Details are provided in the Supplementary Materials.

## 3. Materials and Methods

### 3.1. Chemicals

All reagents: acetylacetone (acacH) (Sigma-Aldrich, St. Louis, MO, USA), Cu(NO_3_)_2_·2.5H_2_O(Sigma Aldrich, St. Louis, MO, USA), and 4,4′-dimethyl-2,2′-bipyridine (dmbpy) (Sigma Aldrich, St. Louis, MO, USA), as the organic solvents, were used without further purification. L-cysteine (Cys) (Sigma Aldrich, St. Louis, MO, USA) was also used without further purification. The elemental analysis of the white crystalline powder for C_3_H_7_O_2_S was %C 29.82 (29.73), %H 5.92(5.82), %N 11.70 (11.56), %S 26.97 (26.46) ((#) calculated values). The far FTIR-ATR spectrum of a deep white powder of Cys showed characteristic bands at 1614 cm^−1^ CO_2_, 1063 cm^−1^ S-H, 692 cm^−1^ C-S, and 637 cm^−1^ CH-CO_2_.

### 3.2. CasIII-ia Synthesis

[Cu(4,4′-dimethyl-2,2′-bipyridine)(acetylacetonate)]NO_3_*(H_2_O)_2_ (CasIII-ia) CAS [223930-33-4], the copper(II) complex, was prepared following the reported patent [31,32,33]. The complex was isolated on MeOH/H_2_O solution, and a blue crystalline powder was obtained. The elemental analysis of the blue powder for CuC_17_H_19_N_3_O_5_*(H_2_O)_2_ was %C 44.26 (45.89), %H 4.79(5.21), and %N 9.45 (9.44) ((#) calculated values). The far FTIR-ATR spectrum of a deep blue powder of CasIII-ia showed characteristic bands at 1616 cm^−1^ C=O (acac), 1373 cm^−1^ N-O (nitrate), 596 cm^−1^ Cu-O, and 294 cm^−1^ Cu-N.

### 3.3. Solid-State Reaction

CasIII-ia (30 mg, 0.0674 mmol) and cysteine (8.17 mg, 0.0674 mmol) were milled until a homogenous solid was obtained. EtOH (400 μL) was added and mixed until dryness.

### 3.4. Measurements

Powder X-ray diffraction (PXRD) data were collected under ambient conditions on a Rigaku ULTIMA IV diffractometer operated at 160 W (40 kV, 40 mA) for Cu Kα1 (λ = 1.5406 Å).

Electron paramagnetic resonance spectroscopy (EPR) measurements were carried out in a JEOL JES-TE300 spectrometer operated at X-Band mode at a microwave frequency of 9.4 GHz and center field of 300 mT. Solid-state measurements were performed at room temperature, where the samples were placed in a quartz cell. The acquisition and manipulation of spectra were performed using the ES-IPRIT/TE program. The g and hyperfine tensors were determined by fitting the powder spectra using the EasySpin [46] simulation package (Version 5.2.28, easyspin.org, (accessed on 6 May 2020)) for MATLAB R2019b.

The solid-state electronic spectra (UV-Vis-NIR) for the samples were measured over the range 40,000–5000 cm^−1^ by the diffuse reflectance method on a Cary-5000 Varian spectrophotometer at room temperature.

The near-FTIR attenuated total reflectance (ATR) spectra were obtained over the range 4000–250 cm^−1^ on a Thermo Fisher Scientific Nicolet IS-50 spectrophotometer. The samples were examined as solid. The middle-FTIR spectra were obtained over the range 4000–400 cm^−1^ on a Nicolet spectrophotometer Nicolet AVATAR 320. The samples were analyzed as KBr disk.

The MS-DART spectra were acquired with a JEOL AccuTOF JMS-T100LC spectrometer. The samples were examined as solid. The values of the signals are expressed in mass/charge units (m/Z), followed by the relative intensity with reference to a 100% base peak.

The elemental analysis (EA) was carried out using The PerkinElmer^®^ 2400 Series II CHNS/O Elemental Analyzer.

### 3.5. Computational Details

All structures were fully optimized at the DFT level in gas phase, with the m05-2x functional and the LanL2DZ basis set, as implemented in Gaussian 09 software [47]. Then, we performed frequency calculations to verify the equilibrium states and to obtain infrared spectra. We used the TD-DFT CAM-B3LYP/SDD theoretical level for the UV-Vis spectra, with the SMD solvation model, with water as the solvent. For the electron transfer studies, we used the Quantum Theory of Atoms In Molecules [48], using the set of molecular orbitals of each molecule to compute the atomic properties of the electron density with the AIMAll software [49]. EPR parameters and g and A tensors of the optimized structures were calculated with ORCA software [50], using the B3LYP functional and the def2-SVP basis set.

The initial complex presented a square planar geometry with a water molecule in one axial site, as in the reported crystallographic geometry [51]. Then, a cysteine molecule was linked to the complex by the vacant axial site, yielding an octahedral geometry. A base in the environment removes the -SH proton, expelling the water molecule from the opposite side. We detected the electron transfer by the complex change to a tetrahedral geometry, which is the geometry preferred by a copper atom with a 1+ oxidation state.

## 4. Conclusions

**Figure 9:**
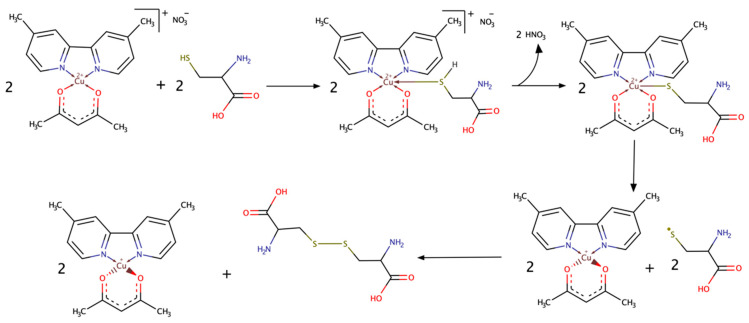
Proposed mechanism for the interaction between CasIII-ia and cysteine.

A strategy to improve cancer therapies’ efficacy should involve cytosolic and mitochondrial GSH depletion through an increase of GSH efflux out of cells. There are reports of GSH depletion produced by copper complexes of the Casiopeina family. In this work, we presented experimental evidence of the formation of an adduct between cysteine and a Casiopeina complex. This adduct shows equilibrium between octahedral and square planar pyramid structures. From this equilibrium, it is possible to identify an electron transfer path when the Cu–S distance increases to 3.9 Å, which produces a thiyl radical and a reduced tetrahedral copper(I) complex. The proposed mechanism is presented in Figure 9.

## Figures and Tables

**Table 1:** Experimental and computational values for the g tensor and the A tensor (in MHz) parameters of spectra A and B in Figure 5.

CasIII-ia (A)			
	Experimental		Computational
g_xx_ = g_yy_	2.0767		2.0466
g_zz_	2.2517		2.1458
A_xx_ = A_yy_	7.47		105.26
A_zz_	117.4		−185.99
**CasIII-ia–Cysteine 1:1 (B)**			
	**Experimental**	**Octahedral**	**Square Planar Pyramid**
g_xx_ = g_yy_ = g_zz_	2.10557	2.1010	2.0755
A_xx_ = A_yy_ = A_zz_	48.797	104.57	23.85

## Data Availability

Data is contained within the article and Supplementary Material.

## Supplementary Materials

The following are available online, Video S1: Reaction in solution. Experimental Details: Figure S1: Powder X-ray diffraction, Table S1: Powder X-ray diffraction (PXRD) data, Table S2: Experimental EPR parameters, Figure S2: Details of the CasIII-ia:Cysteine (1:1) EPR simulation, Figure S3: Deconvolved UV-Vis spectra, Table S3: Comparison between deconvolved and computational values for UV-Vis transitions, Figures S4–S7: Details of ATR-FTIR spectra, Table S4: Main absorption infrared signals, Figures S8 and S9: MS-DART spectra for reactants, computational calculations: optimized geometries, Figures S10–S14: UV-Vis spectra, Figures S15–S17: Infrared spectra, Tables S5–S8: Infrared signals, Table S9: Calculated EPR parameters, Figure S18: Spin density changes, Table S10: Spin density values for Cu and S atoms, Figure S19: Spin density changes in Cu-S bond distance scan, Figure S20: Geometries in the Cu–S bond distance scan, Figures S21 and S22: Copper Atomic graphs.

## Sample Availability

Samples of the compounds are available from the authors.
