# Supplementary material for: Intermediate Detection in the Casiopeina–Cysteine Interaction Ending in the Disulfide Bond Formation and Copper Reduction
Source: Molecules. 2021 Sep 22;26(19):5729. doi: 10.3390/molecules26195729 (PMC8510402; doi:10.3390/molecules26195729)
Supplement: Supplementary file 1 [file molecules-26-05729-s001.zip › molecules-1314545-supplementary.pdf]

Intermediate detection in the Casiopeina-Cysteine Interaction ending in the disulfide bond formation and copper reduction

Lillian G. Ramírez-Palma [1,2] , Adrián Espinoza-Guillén [3], Fabiola Nieto-Camacho [3], Alexis E. López-Guerra [3], Virginia Gómez-Vidales [1] , Fernando Cortés-Guzmán 1,2,* and Lena Ruiz-Azuara [3,*]

[1] Instituto de Química, Universidad Nacional Autónoma de México. Av. Universidad 3000, C. U., México City, 04510, Mexico; lila.gis.rp@gmail.com (L.G.R.P), gomvidal@unam.mx (V.G.V)
[2] Centro Conjunto de Investigación en Química Sustentable UAEMex-UNAM. Carretera Toluca-Atlacomulco km 14.5, Toluca, 50200, Mexico.
[3] Facultad de Química, Universidad Nacional Autónoma de México. Av. Universidad 3000, C. U., México City, 04510, Mexico; adrianeg24@gmail.com (A.E.G); fabille.nc@gmail.com (F.N.C.); eduga_9@live.com.mx (A.E.L.G)
* Correspondence: fercor@unam.mx (F.C.G.); lenar701@gmail.com (L.R.A)

Supplementary Materials

Experimental Section

1. Experimental Details

Chemicals

All reagents: acetylacetone (acacH) (Sigma), $Cu(NO_3)_2$ (Aldrich), 4,4'-dimethyl-2,2'-bipyridine (dmbpy) (Aldrich), as the organic solvents, were used without further purification. L-cysteine (Cys) (Aldrich) was used without further purification. The elemental analysis of the white crystalline powder for $C_3H_7NO_2S$ was %C 29.82 (29.73), %H 5.92(5.82), %N 11.70 (11.56) % S 26.97 (26.46)[(#) calculated values]. The far FTIR-ATR spectrum of a deep white powder of cys showed characteristic bands at 1614 cm^{-1} CO_2, 1063 cm^{-1} S-H, 692 cm^{-1} C-S, 637 cm^{-1} $CH-CO_2$.

CasIII-ia Synthesis

[Cu(4,4'-dimethyl-2,2'bipyridine)(acetylacetonate)]NO_3*$(H_2O)_2$ (CasIII-ia) CAS [223930-33-4]. The copper (II) complex was prepared following the reported patent. The complex was isolated on MeOH/H_2O solution; a blue crystalline powder was obtained. The elemental analysis of the blue powder for $CuC_{17}H_{19}N_3O_5$*$(H_2O)_2$ was %C 44.26 (45.89), %H 4.79(5.21), %N 9.45 (9.44) [(#) calculated values]. The far FTIR-ATR spectrum of a deep blue powder of CasIII-ia showed characteristic bands at 1616 cm^{-1} C=O (acac), 1373 cm^{-1} N-O (nitrate), 596 cm^{-1} Cu-O, 294 cm^{-1} Cu-N.

Solid-state reaction

CasIII-ia (30 mg, 0.0674 mmol) and cysteine (8.17 mg, 0.0674 mmol) were milled until a homogenous solid was obtained. EtOH (400 µL) was added and mixed up to dryness.

Measurements

Powder X-ray diffraction (PXRD) data were collected under ambient conditions on a Rigaku ULTIMA IV diffractometer operated at 160 W (40 kV, 40 mA) for Cu Kα1 (λ= 1.5406 Å).

Electron Paramagnetic Resonance Spectroscopy (EPR) measurements were carried out in a JEOL JES-TE300 spectrometer operated at X-Band mode at microwave frequency of 9.4 GHz and center field 300 mT. Solid-state measurements were made at room temperature where the

samples were placed in a quartz cell. The acquisition and manipulation of spectra were performed using the ES-IPRIT/TE program. The g and hyperfine tensors were determined by fitting the powder spectra using EasySpin[1] simulation package (Version 5.2.28, easyspin.org) for MATLAB R2019b.

The solid-state electronic spectra (UV-Vis-NIR) for the samples were measured over the range 40000-5000 cm^{-1} by the diffuse reflectance method on a Cary-5000 Varian spectrophotometer at room temperature.

The Near-FT-IR attenuated total reflectance (ATR) spectra were obtained over range 4000-250 cm^{-1} on a Thermo Fisher Scientific Nicolet IS-50 spectrophotometer. The samples were examined as solid. The Middle-FT-IR spectra were obtained over range 4000-400 cm^{-1} on a Nicolet spectrophotometer Nicolet AVATAR 320. The samples were examined as KBr disk.

The MS-DART were acquired with an JEOL AccuTOF JMS-T100LC spectrometer. The samples were examined as solid. The values of the signals are expressed in mass/charge units (m/Z), followed by the relative intensity whit reference to a 100% base peak.

The elemental analysis (EA) was carried out in the Research and Industry Support Services Unit-UNAM (USAII-UNAM for its acronyms Spanish), using The PerkinElmer® 2400 Series II CHNS/O Elemental Analyzer.

1. *Powder X-ray diffraction (PXRD)*

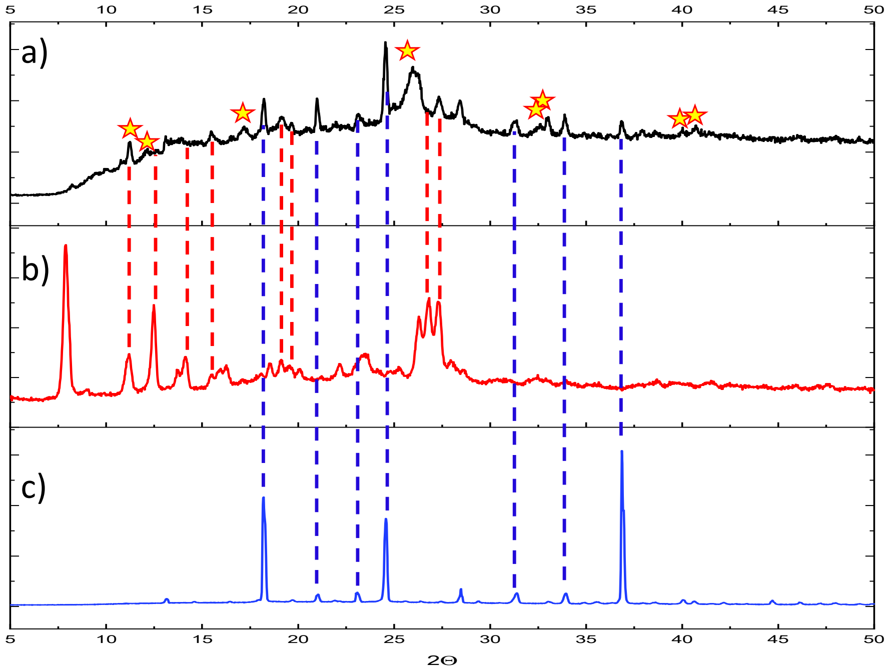

Figure S1. Powder X-ray diffraction (PXRD) a) CaIII-ia:Cys 1:1 were new signals are presented as symbol star, b) CasIII-ia and c) cysteine.

Cysteine	CasIII-ia	CasIII-ia:Cys 1:1
2θ	2θ	2θ

13.16	7.9	8.22
18.18	11.2	10.76
21.04	12.48	11.24
23.02	13.74	12.12
23.08	14.12	13.1
24.56	15.46	15.46
28.48	15.96	17.18
31.38	16.26	18.22
36.86	18.54	19.14
40.06	19.12	19.68
40.58	19.56	20.98
40.66	20.08	23.08
	22.18	24.54
	23	25.94
	23.32	27.32
	23.64	28.42
	25.22	31.38
	26.28	32.58
	26.82	33.02
	27.28	33.88
	27.94	36.84
	28.56	37.92
		38.58
		40.02
		40.72

Table S1. Powder X-ray diffraction (PXRD) data.

2. *Electron Paramagnetic Resonance*

	CasIII-ia	*Solid-state reaction* CasIII-ia:Cysteine 1:1	
v [MHz]	9439.6	9434.57	
Symmetry	Axial	Axial (1)	Isotropic (3)
$g_{xx} = g_{yy}$	2.0767	2.07884	
g_{zz}	2.2517	2.30167	
$g_{xx} = g_{yy} = g_{zz}$			2.10557
**$A_{xx} = A_{yy}$ [MHz]**	7.47	6.735	
**$A_{xx} = A_{yy}$ [mT]**	0.267	0.240	
**$A_{xx} = A_{yy}$ [$x10^{-4}$ cm^{-1}]**	2.49	2.25	
**A_{zz} [MHz]**	117.4	118.135	
**A_{zz} [mT]**	4.189	4.215	
**A_{zz} [$x10^{-4}cm^{-1}$]**	39.16	39.41	

**$A_{xx}=A_{yy}=A_{zz}$ [MHz]**	48.797
**$A_{xx}=A_{yy}=A_{zz}$ [mT]**	1.741
**$A_{xx}=A_{yy}=A_{zz}$ [$x10^{-4}cm^{-1}$]**	16.27

Table S2. Experimental EPR parameters.

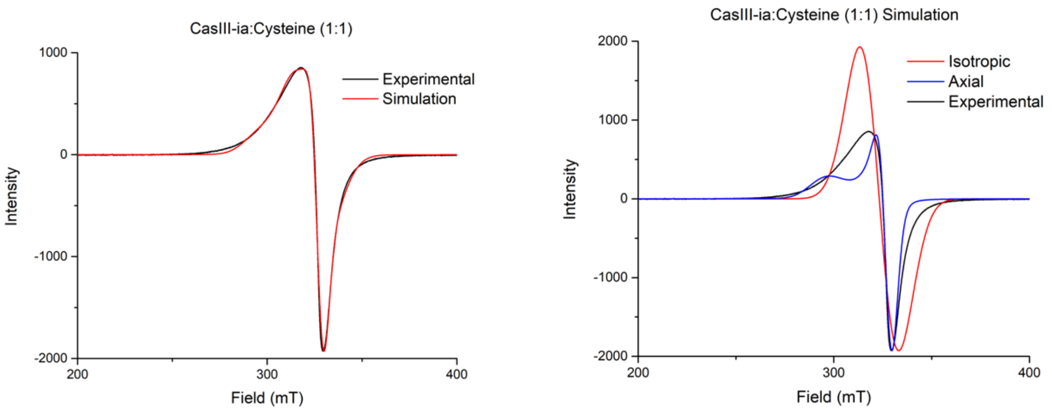

Figure S2. Details of the CasIII-ia:Cysteine (1:1) EPR simulation. Left: Final spectrum. Right: Isotropic and axial species with a relation 3:1, respectively, considered for the final spectrum simulation.

3. *Electronic spectra (UV-Vis-NIR)*

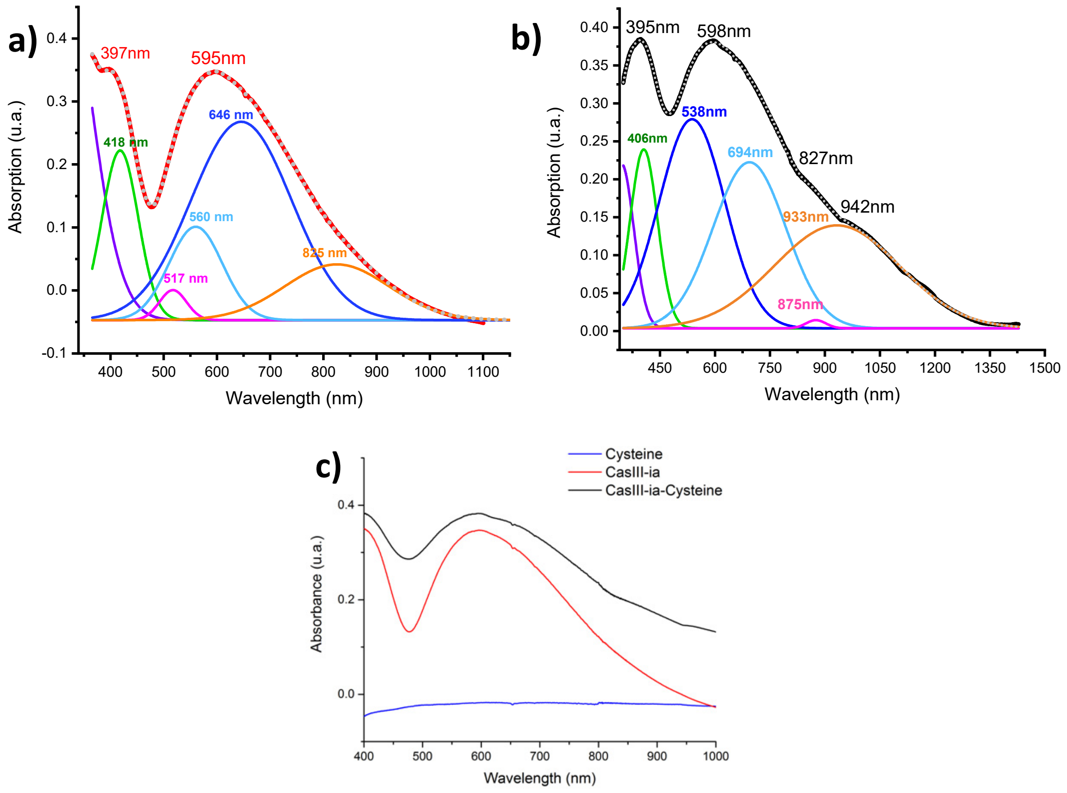

Figure S3. (a) Deconvolved experimental spectrum for CasIII-ia; (b) Deconvolved experimental spectrum for the CasIII-ia:Cysteine (1:1) reaction; (c) Comparison between reactants and intermediate.

CasIII-ia			
Experimental	Deconvolution	Computational	
397	418	414	
595	517	523	
	560	581	
	646	615	
	825	826	
CasIII-ia-Cysteine (1:1)			
		Computational	
Experimental	Deconvolution	Octahedral	Square planar pyramid
395	406	400	398
598	538	553	
	694	647	654
	875		
	933	1077	955

Table S3. Comparison between deconvolved and computational values for UV-Vis transitions, for CasIII-ia and the reaction CasIII-ia:Cysteine (1:1) with octahedral and square planar pyramid computational geometries.

4. *Infrared spectroscopy FT-IR*

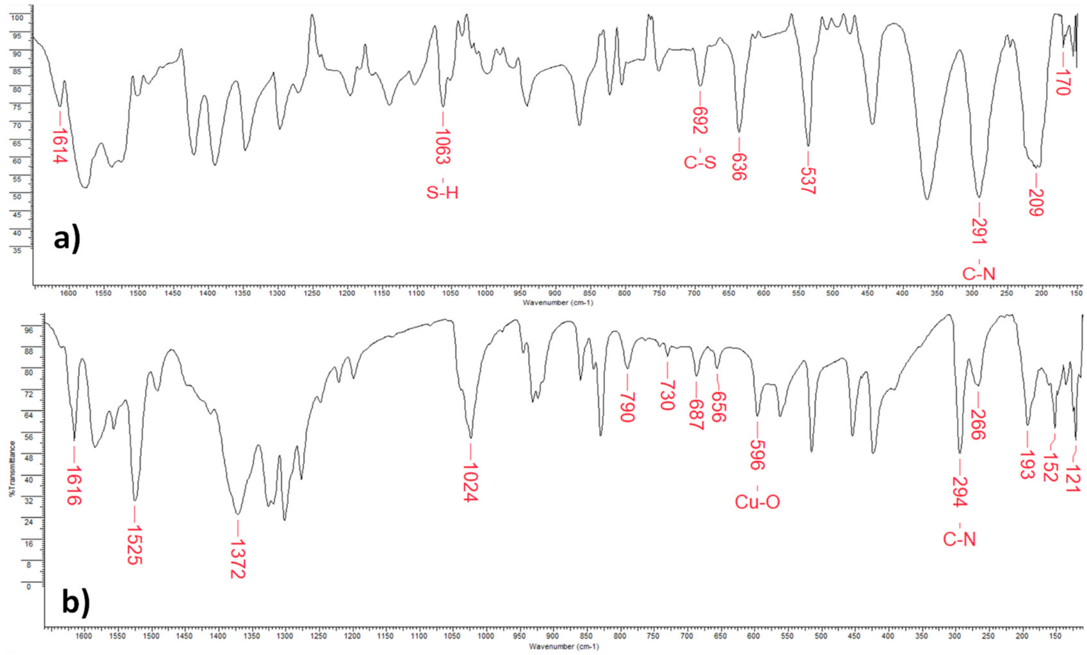

Figure S4. ATR-FTIR spectrum of (a) cysteine and (b) CasIII-ia.

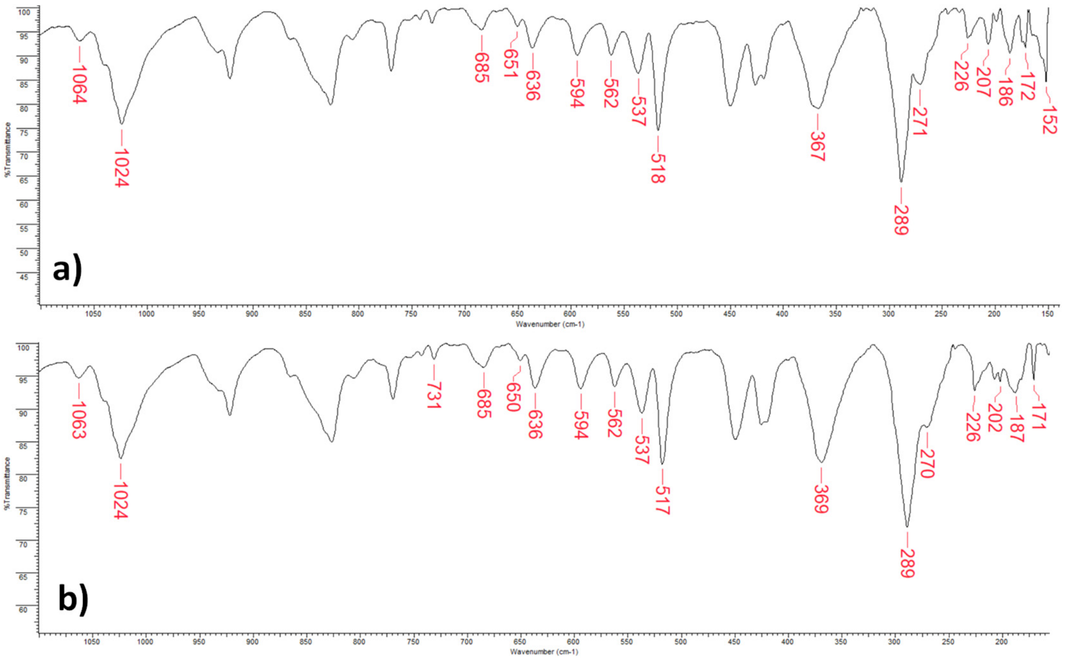

Figure S5. ATR-FTIR spectrum of the mixtures CasIII-ia-Cys 1:1: reaction between CasIII-ia and Cysteine with a proportion 1:1 at (a) 5 and (b) 20 minutes.

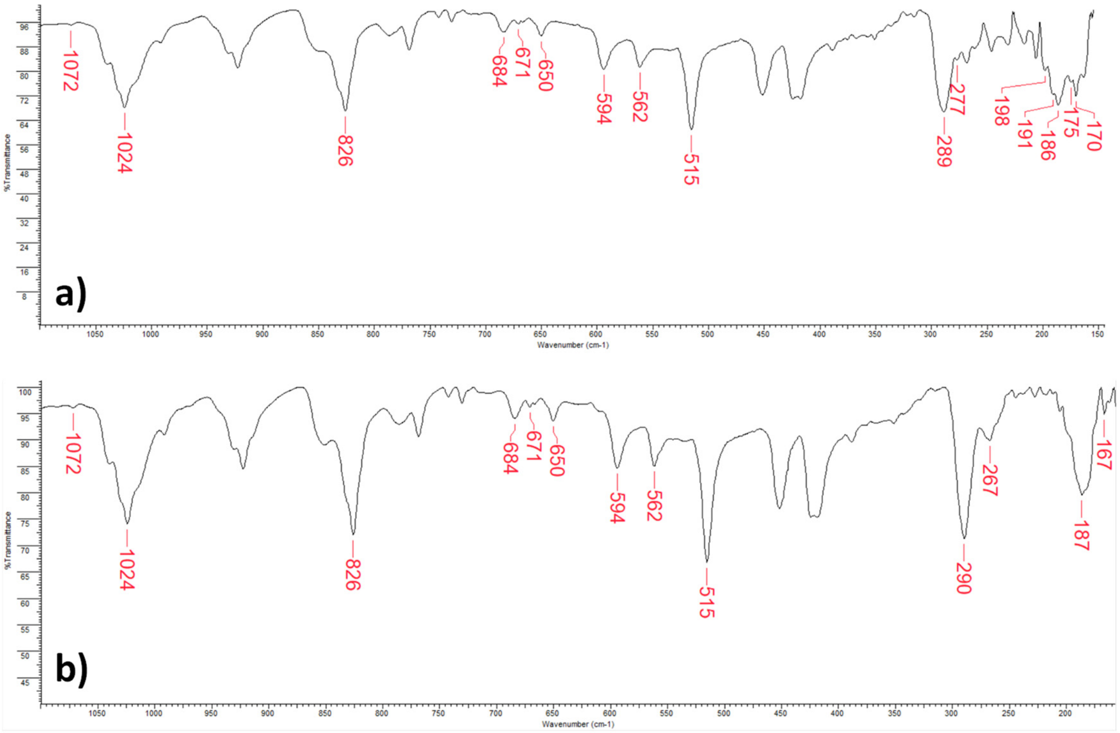

Figure S6. ATR-FTIR spectrum of CasIII-ia-Cys-OH: deprotonation of cysteine, after mixing with CasIII-ia, by adding NaOH, with a proportion 1:1:1 at (a) 5 and (b) 20 minutes.

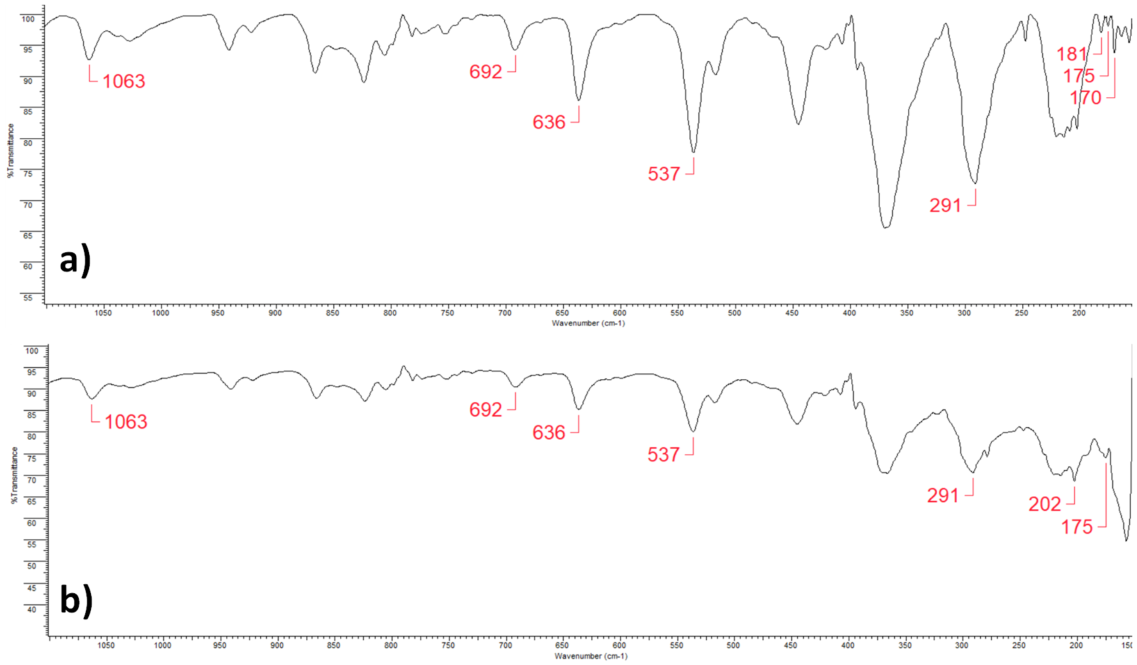

Figure S7. ATR-FTIR spectrum of CasIII-ia-Cys 1:5 reaction between CasIII-ia and Cysteine with a proportion 1:5 at (a) 8 and (b) 20 minutes.

**Signal**	**Cys**	**Cas**	**Cas-Cys 1:1**		**Cas-Cys-OH**		**Cas-Cys 1:5**	
			5 min	***20 min***	***5 min***	***20 min***	***8 min***	***20 min***
**Cu-S** $_{str}$	—	—	186	187	186	187	181	175
**Cu-S** $_{as}$	—	—	271	270	268	267	—	279
**C-N**	291	294	289	289	—	290	291	291
**Cu-O**	—	596	593	594	594	594	—	599
**C-S**	636	—	651	650	650	650	636	636
**C-S**	692	—	—	—	—	707	692	692
**S-H**	1063	—	1064	1063	1072	1072	1063	1063

Table S4. Main absorption infrared signals through ATR for the mixtures: (a) CasIII-ia-Cys 1:1: reaction between CasIII-ia and Cysteine with a proportion 1:1; (b) CasIII-ia-Cys-OH: deprotonation of cysteine, after mixing with CasIII-ia, by adding NaOH, with a proportion 1:1:1; (c) CasIII-ia-Cys 1:5: reaction between CasIII-ia and Cysteine with a proportion 1:5. The time indicated for every measurement was counted from the addition of the solvent.

5. *Mass Spectra-Direct Analysis in Real Time (MS-DART)*

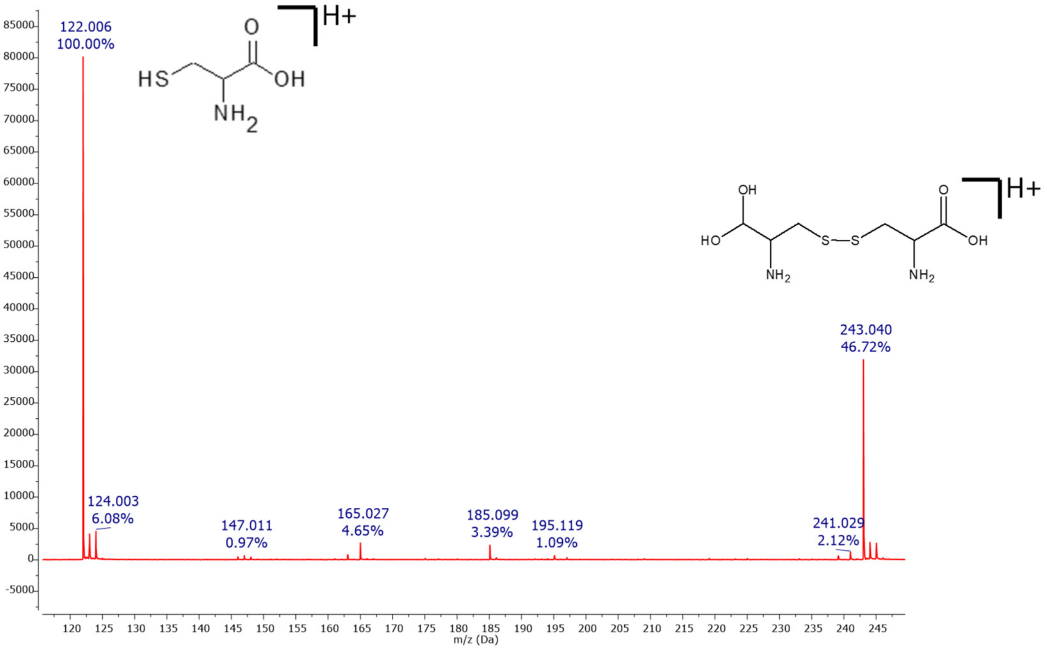

Figure S8. MS-DART spectrum for Cysteine.

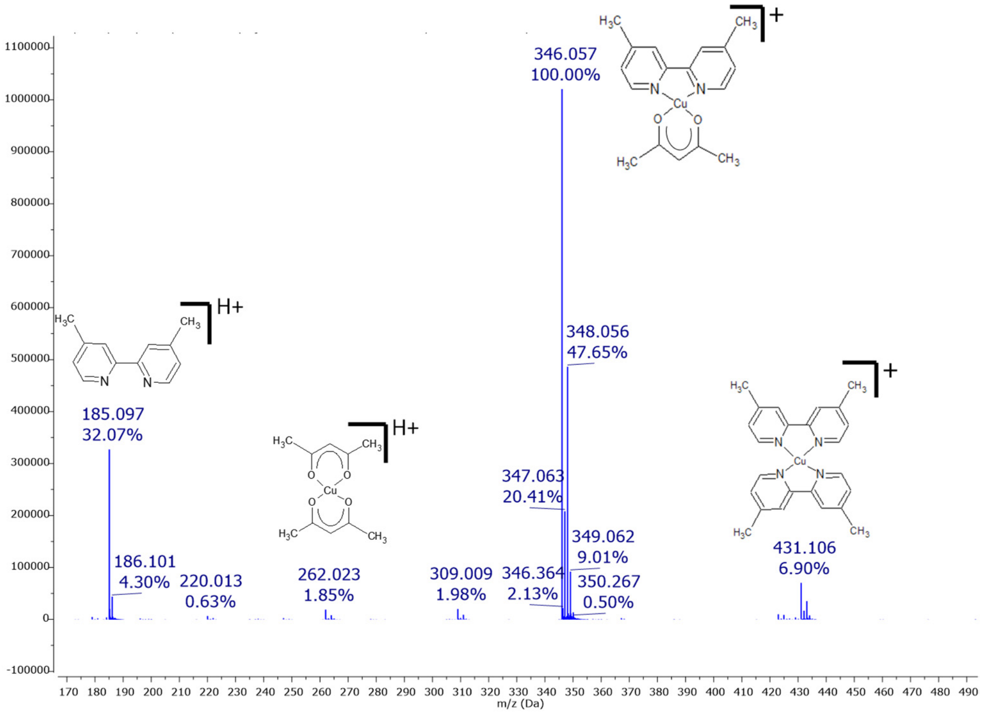

Figure S9. MS-DART spectrum for CasIII-ia.

Computational Section

1. Computational Geometries

All structures were fully optimized at DFT level, with the m05-2x functional and the LanL2DZ basis set as implemented in Gaussian 09 software[2]. Then we performed frequency calculations to verify the equilibrium states and to obtain Infrared spectra. For the UV-Vis spectra, we used the TD-DFT CAM-B3LYP/SDD theoretical level, with the SMD solvation model with water as the solvent. For the electron transfer studies, we used the Quantum Theory of Atoms In Molecules[3], using the set of molecular orbitals of each molecule to compute the atomic properties of the electron density with the AIMAll software[4]. EPR parameters, g and A tensors, of the optimized structures were calculated with ORCA software[5], using the B3LYP functional and the def2-SVP basis set.

a) Cysteine

Energy: -333.706254 u.a.

0 1

C	-0.15754200	1.25852000	0.03985800
C	-1.43934200	2.02288500	0.39046900
H	-0.06362100	1.19664800	-1.04176100
H	0.70693200	1.75361200	0.47979300
N	-1.77352500	2.03471400	1.82153600
H	-1.31020500	3.04817600	0.03273600
C	-2.62206500	1.45311000	-0.42649500
H	-1.73571500	2.93812800	2.27385700
O	-3.71973000	1.19805900	0.32803600
H	-1.33595900	1.29473200	2.36248200
O	-2.56252500	1.28421200	-1.64305800
S	-0.28168000	-0.48996800	0.74217100
H	-3.48805500	1.41377500	1.27385600
H	1.03155400	-0.83881000	0.55942900

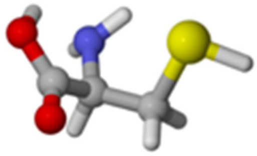

b) Casiopeina (CasIII-ia)

Energy: -1191.447032 u.a.

1 2

Cu	0.84319900	0.01223000	0.10262300
O	2.15717700	1.41392500	-0.13189800
O	2.18878200	-1.37560000	-0.03634600
N	-0.68930700	1.31011200	-0.01459100
C	-1.92057100	0.73959400	-0.07029400
C	-1.91160200	-0.74592500	-0.09037700
N	-0.67410800	-1.30175600	-0.07882200
C	-0.52751000	-2.63972900	-0.11285700
C	-1.63536700	-3.48939700	-0.15740700
C	-2.93107400	-2.94457400	-0.16738600
C	-3.05584800	-1.54310900	-0.13110100
C	-3.07138600	1.52492800	-0.12814700
C	-2.96225500	2.92898600	-0.12966900
C	-1.67406800	3.48817200	-0.08177100
C	-0.55713900	2.65023400	-0.02542600
H	0.49109600	-3.00021500	-0.10632200
H	-1.48850700	-4.56057600	-0.18439000
H	-4.04045900	-1.09629500	-0.13547300
H	-4.04984300	1.06731700	-0.18018800
H	-1.53842400	4.56094900	-0.09366200
H	0.45769300	3.02064600	-0.00080400
C	4.27155700	-2.48498300	-0.36649100
H	5.34158300	-2.29297900	-0.41908700
H	4.05780600	-3.13776500	0.48277400
H	3.96019000	-3.01371500	-1.27179000
C	4.21847900	2.55209600	-0.48231100
H	4.01144600	3.21878200	0.35793300
H	5.29024900	2.37610500	-0.55155700
H	3.87865800	3.05559400	-1.39141900
O	0.96976300	-0.13442400	2.29541300
H	0.85382000	0.55492500	2.97016500
H	1.49910200	-0.88907000	2.60644300
C	3.47359300	-1.21576600	-0.24190400
C	4.10810900	0.03277600	-0.35754800
H	5.17522600	0.04071900	-0.51806000
C	3.44379300	1.27443800	-0.30919200
C	-4.14673500	-3.82960500	-0.24159900
H	-5.01872800	-3.35036600	0.20531400
H	-4.38625900	-4.05217900	-1.28579000
H	-3.97591600	-4.77912500	0.26727300
C	-4.19162600	3.79703500	-0.16962800
H	-4.97016000	3.35409900	-0.79306400
H	-4.60151200	3.91738100	0.83768300
H	-3.96380500	4.79037200	-0.55686600

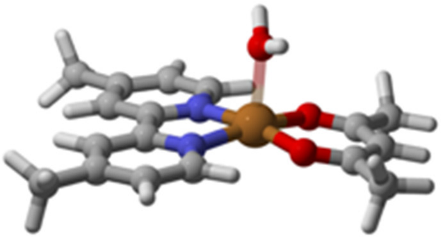

c) Octahedral complex

Energy: -1524.756847 u.a.

0 2

O	-2.63657300	1.47048000	0.06657200
O	-2.49493800	-1.00377100	-1.18623500
C	-4.26005900	-2.55987400	-0.82111200
H	-5.31248900	-2.58634900	-0.54124200
H	-4.13887900	-2.85984900	-1.86341100
H	-3.70834100	-3.27320300	-0.20004700
C	-4.56851900	1.96337900	1.37171300
H	-4.59500900	2.95353700	0.91146600
H	-5.58306400	1.61279700	1.55573300
H	-4.04302700	2.05410400	2.32705500
C	-3.64600100	-1.20480900	-0.60534500
C	-4.30498900	-0.25163600	0.19807500
H	-5.25340900	-0.52884600	0.63277800
C	-3.79040200	1.01890000	0.49583500
Cu	-1.15807500	0.30083600	-0.53144500
S	-1.05736800	-1.27104500	1.63655200
C	0.19351300	-1.24740400	3.06920800
C	1.62011500	-0.82811900	2.68472700
H	0.19861900	-2.25533400	3.48853300
H	-0.17169800	-0.55387100	3.82805700
N	1.61740400	0.51391200	2.10252200
H	2.24195900	-0.84142300	3.58618800
C	2.32647700	-1.77812300	1.70059400
H	0.69922600	0.87683200	1.88472000
H	2.32002300	1.16544200	2.41300700
O	1.54280900	-2.53833200	0.89589200
O	3.56343900	-1.82730500	1.61412000
N	0.20424900	1.77229200	-0.37471600
C	1.44733900	1.41616400	-0.77601300

C	-0.04696100	3.02472300	0.04790700
C	1.57736600	0.01056000	-1.22074800
C	2.49070100	2.34423000	-0.78166800
C	0.95884800	3.99246000	0.08137000
H	-1.06487200	3.21914400	0.35545800
N	0.39671300	-0.63129800	-1.40374600
C	2.79087600	-0.64320300	-1.41410800
C	2.25658600	3.65773900	-0.34662300
H	3.47605500	2.05202900	-1.11842400
H	0.73262900	4.99214800	0.42745500
C	0.37865200	-1.89356900	-1.86633200
C	2.79575000	-1.98266200	-1.84008100
H	3.72436000	-0.15629800	-1.16792400
H	-0.59930600	-2.33941800	-1.97871100
C	1.56245700	-2.58772800	-2.12346900
H	1.51837700	-3.61445200	-2.45992500
O	-1.98939800	1.40630000	-2.73453100
H	-2.52115100	1.85273300	-2.04657900
H	-2.31486800	0.49698400	-2.87828600
C	4.08139200	-2.75893600	-1.87401800
H	4.33059100	-3.03036600	-0.84231000
H	3.98813400	-3.67239300	-2.46300400
H	4.90436300	-2.16197100	-2.27336500
C	3.36013400	4.68482200	-0.36523000
H	3.15562900	5.49893200	0.33173900
H	4.32050900	4.23704400	-0.10285000
H	3.45631600	5.11620900	-1.36614500
H	0.54743700	-2.30773500	0.96597700

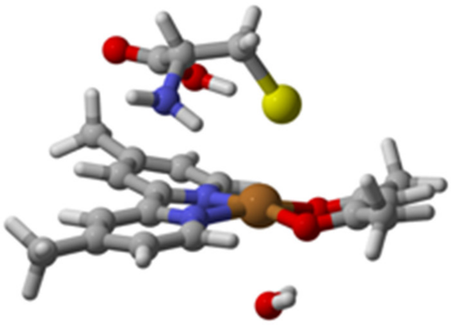

d) Square planar pyramid complex

Energy: -1524.760727 u.a.

0 2

O	-2.23379100	-0.32998200	-0.02734200
O	-0.41728100	1.50601300	1.11143700
C	-1.10697700	3.78640200	1.22249200
H	-2.01171700	4.37876100	1.35668300
H	-0.47577900	3.85585900	2.11071500
H	-0.54338400	4.19553600	0.37499300
C	-4.47127600	0.41771800	-0.40019800
H	-4.85573000	-0.44947200	0.14301300
H	-5.11908600	1.27528700	-0.22390900
H	-4.48032000	0.17449100	-1.46648500
C	-1.40778900	2.34381100	0.91779700
C	-2.67553900	1.98037400	0.43695000
H	-3.41999300	2.75962800	0.36080100
C	-3.04642800	0.67990100	0.02737100
Cu	-0.10414500	-0.05974200	-0.06197400
S	0.26476300	1.36564700	-1.92516400
C	2.15026900	1.38354700	-1.98394100
C	2.75684500	2.44389400	-1.05458100
H	2.43344600	1.61996700	-3.01027100
H	2.54068200	0.39641000	-1.71866800
N	2.35614000	2.36542100	0.35895000
H	3.84895300	2.36530400	-1.10863400
C	2.42525200	3.85643600	-1.58102000
H	3.01878600	1.90900200	0.97184400
H	1.38984800	2.06967000	0.51942500
O	2.05801800	4.71037700	-0.58305200
O	2.52918500	4.18732200	-2.76123900
N	0.47567100	-1.79574000	-0.96735600
C	0.65147600	-2.85625000	-0.13928400
C	0.59598400	-1.95437300	-2.29886600
C	0.44522800	-2.56833000	1.29987000
C	0.99022200	-4.11253900	-0.64010400
C	0.91819100	-3.19378200	-2.86094200
H	0.42677900	-1.06297600	-2.89021400
N	0.16513900	-1.27576600	1.57800900
C	0.50024800	-3.52690200	2.31254200
C	1.12663900	-4.30132600	-2.02566800
H	1.13170800	-4.94943000	0.02995500
H	1.00554500	-3.28462400	-3.93528800
C	-0.08420300	-0.88944900	2.84111000
C	0.24876000	-3.14939800	3.64114600
H	0.70600400	-4.56290300	2.08144000
H	-0.31623000	0.15793600	2.97026700
C	-0.04580800	-1.80034500	3.89973800
H	-0.25172300	-1.46340400	4.90693900

H	2.08841900	4.16410900	0.25960400
O	-2.23626800	-2.99052700	0.22123100
H	-2.79743300	-3.46776300	-0.41005400
H	-2.33146200	-2.00452800	0.13417300
C	1.47047300	-5.65836800	-2.58549600
H	2.36420800	-6.06361400	-2.10480200
H	0.65231400	-6.36257100	-2.41052500
H	1.65171600	-5.60947200	-3.65962200
C	0.25434700	-4.17392300	4.74706800
H	-0.70591100	-4.69736400	4.77665600
H	1.03565200	-4.92078300	4.59190700
H	0.41128600	-3.70626900	5.72016200

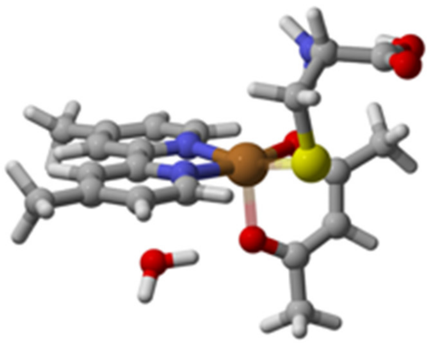

2. *Spectroscopy*

UV-Vis

For the UV-Vis spectra, we used the TD-DFT CAM-B3LYP/SDD theoretical level, with the SMD solvation model with water as the solvent implemented in Gaussian09.

a) Cysteine

Figure S10 shows the absorption spectrum of the cysteine molecule. There are 3 absorption bands at 130, 150 and 180 nm.

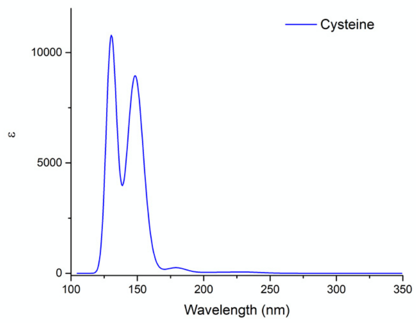

Figure S10. UV-Vis spectra for cysteine.

b) Casiopeina (CasIII-ia)

Figure S11 shows the absorption spectrum of the Casiopeina III-ia (composed by acetylacetonate and bipyridine). This spectrum shows the mixed transitions of the aromatic ligand and the acetylacetonate in a band near to 260 nm. If we made zoom in the figure, we can appreciate the atom copper transitions in a band near to 580 nm.

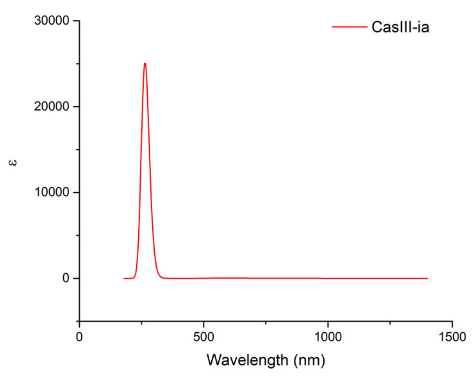

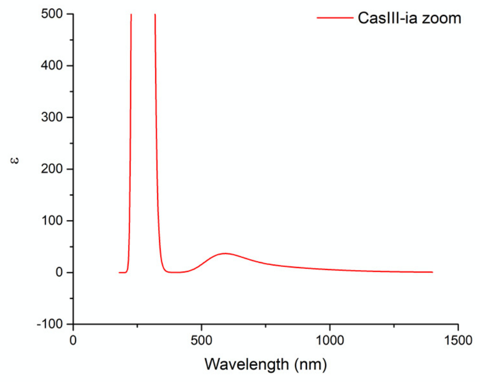

Figure S11. UV-Vis spectra for CasIII-ia.

Molecular Orbital Transitions associated to excited state: 580.82nm, 2.1346 eV, f=0.0007, <S**2>=0.751.

67B -> 91B	-0.11947
76B -> 91B	-0.45981
78B -> 91B	0.12965
80B -> 91B	0.46908
81B -> 91B	-0.57588
81B -> 92B	-0.10526
85B -> 91B	-0.26049
86B -> 91B	0.23162

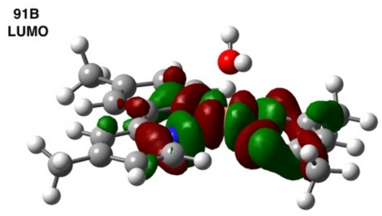

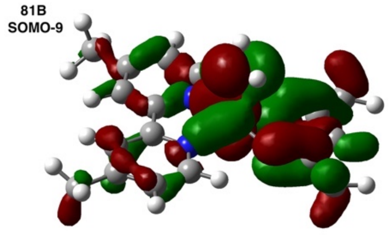

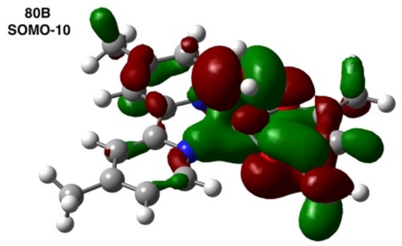

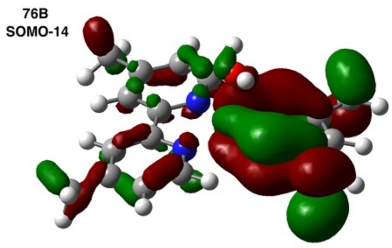

c) Octahedral Complex

Figure S12 shows the absorption spectrum of CasIII-ia with a cysteine molecule coordinated in the free axial position of the copper complex, which gives us an octahedral complex. Now the mixed transitions of the aromatic ligand, the acetylacetonate and cysteine are around 330 nm. If we made a zoom, we can notice that the copper transitions are displaced until 625 nm.

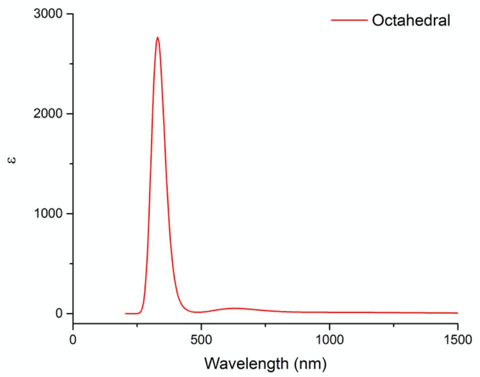

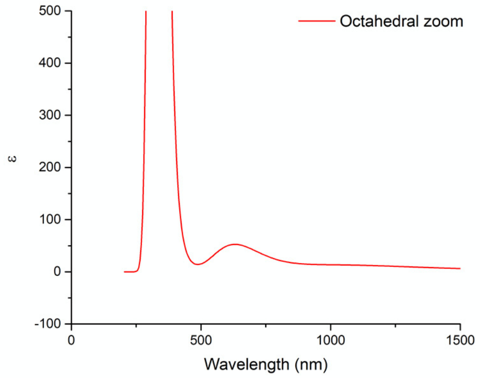

Figure S12. UV-Vis spectra for Octahedral complex.

Molecular Orbital Transitions associated to excited state: 625.82nm, 1.9811 eV, f=0.0010, <S**2>=0.753.

102B ->122B	-0.26811
102B ->123B	-0.19736
102B ->124B	0.15645
102B ->125B	0.10068
104B ->122B	-0.18426
104B ->123B	-0.13729
104B ->124B	0.10531
105B ->122B	-0.10204
106B ->122B	-0.39619
106B ->123B	-0.29173
106B ->124B	0.22572
106B ->125B	0.14557
107B ->122B	-0.25192
107B ->123B	-0.18273
107B ->124B	0.14245

109B ->122B	0.12458
113B ->122B	-0.16381
113B ->123B	-0.11564
114B ->122B	0.10495
116B ->122B	0.20277
116B ->123B	0.14668
116B ->124B	-0.10481

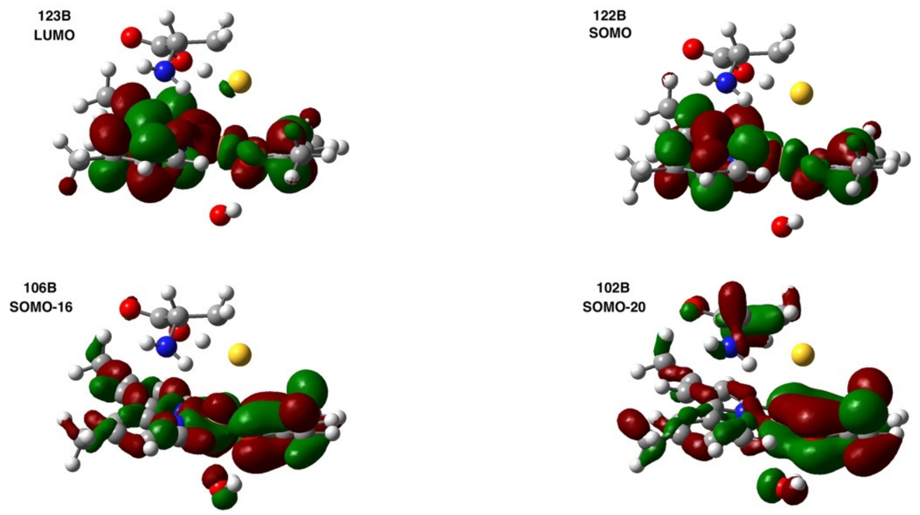

d) Square planar pyramid complex

Figure S13 shows the absorption spectrum for Cas III-ia with a cysteine molecule that removed the non-aromatic ligand from one of the coordination sites, producing a square planar pyramid complex. In this case, the aromatic ligand, acetylacetonate and cysteine transitions are added around 340 nm. In the zoom, the copper transitions are displaced until 663 nm.

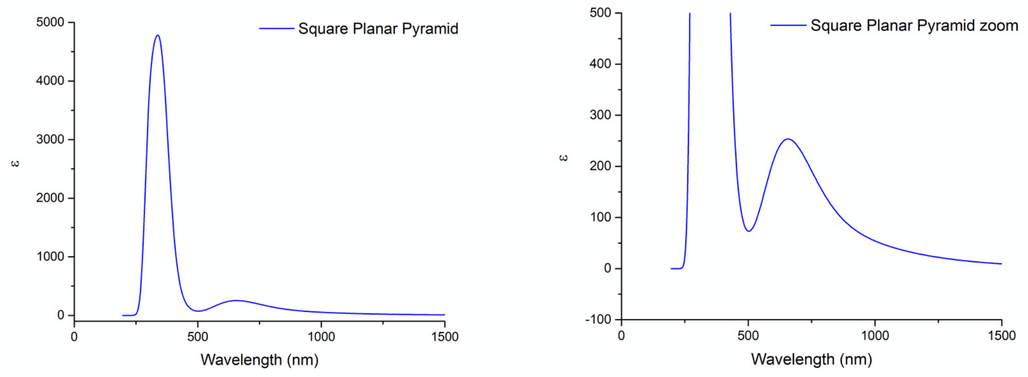

Figure S13. UV-Vis spectra for Square planar pyramid complex.

Molecular Orbital Transitions associated to excited state: 663.40 nm, 1.8689 eV, f=0.0048, <S**2>=0.755.

103B ->122B	-0.22145
103B ->123B	-0.23155
103B ->124B	-0.11246
104B ->122B	-0.29974
104B ->123B	-0.29947
104B ->124B	-0.14387
105B ->123B	0.10031
107B ->122B	-0.27729
107B ->123B	-0.28172
107B ->124B	-0.13213
110B ->122B	-0.11772
110B ->123B	-0.11930
111B ->122B	-0.12085
111B ->123B	-0.12429
113B ->122B	0.10387
113B ->123B	0.10847
115B ->122B	-0.19640
115B ->123B	-0.19527
117B ->122B	0.10480
117B ->123B	0.10403
120B ->122B	0.25605
120B ->123B	0.25923

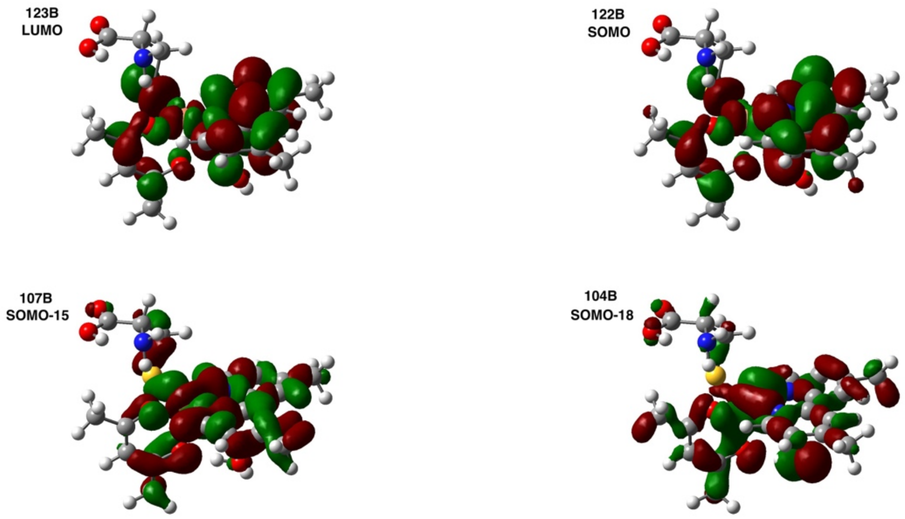

e) Comparison between b, c and d spectra

Figure S14 is the comparison between the CasIII-ia, the octahedral and the square planar pyramid complexes, to appreciate in a better way the displacement of the absorption maxima.

In the case of the copper transition, when the coordination environment changes, the values of the maximum change either. If we remember, the value for the CasIII-ia is 590 nm, for the octahedral complex it is 630 nm and for the square planar pyramid it is 656 nm. We observed a shift of the signal to the right, this change is more drastic when moving to the square planar pyramid complex.

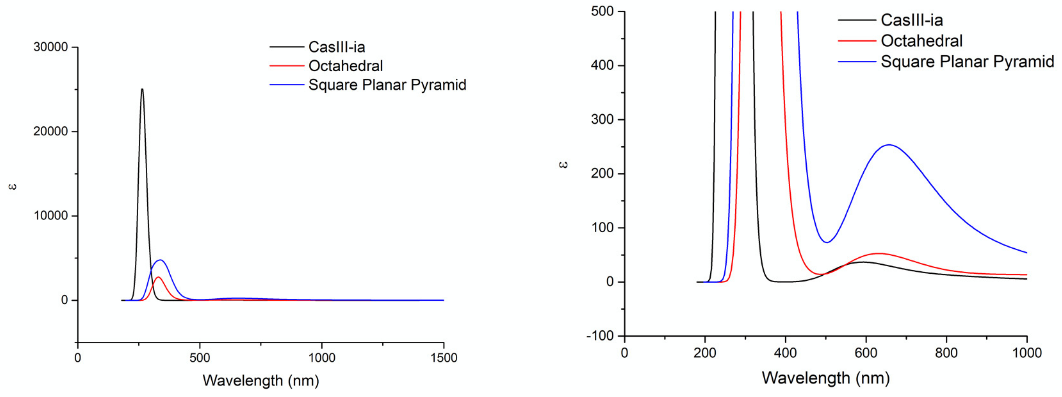

Figure S14. UV-Vis comparison spectra between Cas III-ia, Octahedral and Square planar pyramid complexes.

Infrared

We performed the frequencies calculations at m05-2x/LanL2DZ theory level with the Gaussian09 software. First, we compare the experimental and computational spectra of the CasIII-ia and cysteine. And later, there is the comparison between the experimental reaction and the computational interaction CasIII-ia with cysteine.

a) **Cysteine**

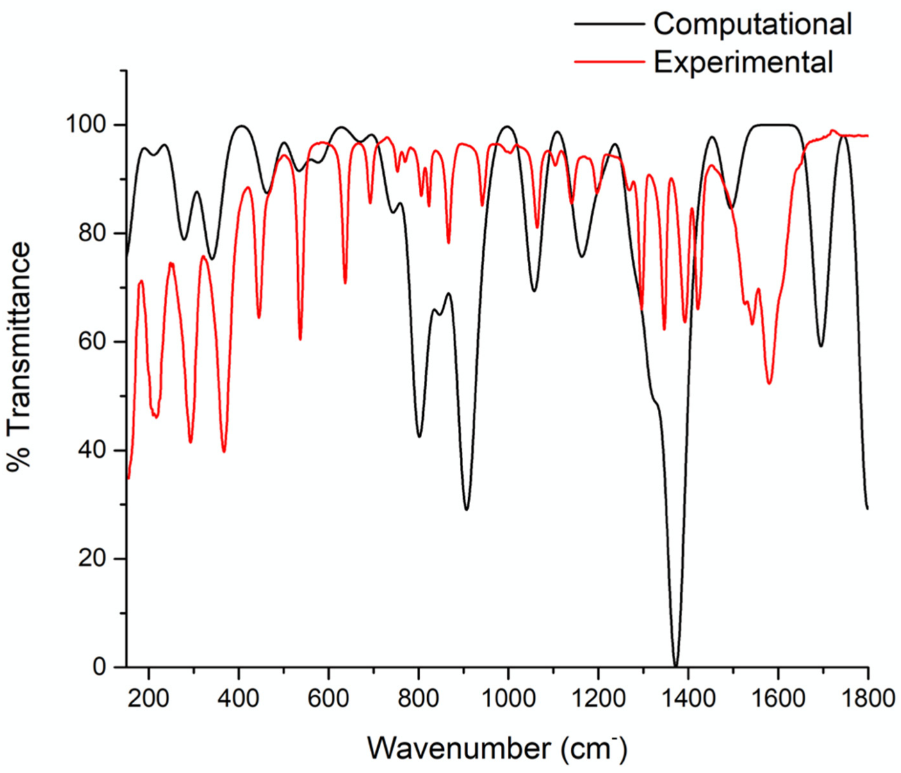

Figure S15. IR spectra for cysteine.

Signal Atoms	Type	Computational	Experimental
C-N	stretch	278	292.6449
C-S	stretch	669.99	692.3198
N-H2	wagging	801.4	
CO2	wagging	848	
N-CH	stretch	943.72	
S-H	in-plane bending	1056.73	1063.55
CH2	twisting	1173.6	
CH	bending	1279.58	
CH2	scissors	1494.82	
NH2	scissors	1695.19	
CO2	asymmetric stretch	1798.67	
S-H	stretch	2605	
C-H	stretch	3122.74	
CH2	symmetric stretch	3154.94	
CH2	asymmetric stretch	3234.24	
NH2	symmetric stretch	3589.41	
NH2	asymmetric stretch	3728.3	

Table S5. Infrared signals for cysteine.

b) Casiopeina III-ia

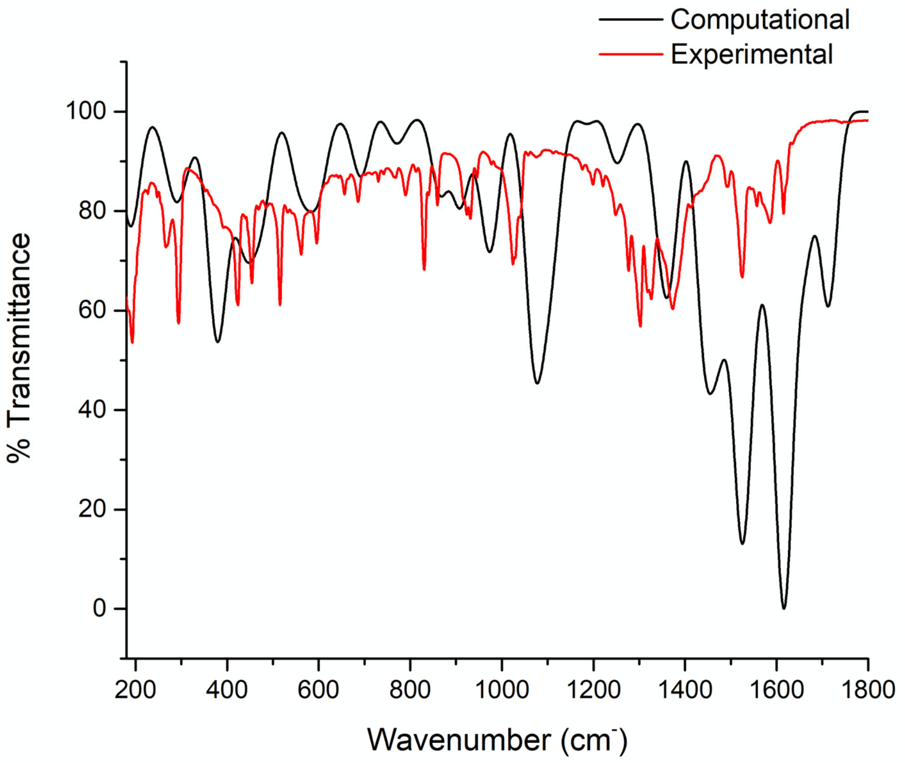

Figure S16. IR spectra for CasIII-ia.

Signal Atoms	Type	Computational	Experimental
**Cu-O**	wagging	192.25	
**C-N aromatic**	twisting	279.73	294
**Cu-N**	scissoring	356.37	
**Cu-O**	Rocking/ asymmetric stretch	423.14	
**Cu-O**	symmetric stretch	474.39	
**Cu-N**	symmetric stretch	581.20	
**Cu-O**	rocking	603.88	596
**Cu-N**	symmetric stretch	1074.60	
**C-O acac**	symmetric stretch	1530.06	
**C-C acac**	asymmetric stretch	1614.63 , 1623.29	
**C-C aromatic**	symmetric stretch	1660.88, 1664.29	
**C-C aromatic**	asymmetric stretch	1711.21, 1716.16	

Table S6. Infrared signals for CasIII-ia.

c) Comparison between theoretical spectrum (black line) and experimental spectrum after 5 minutes (red line) and after 35 minutes (blue line) for the geometries (from top to bottom): (1) protonated octahedral, (2) square planar pyramid and (3) deprotonated octahedral.

We can appreciate that there is not a big change between both experimental spectra (blue and red lines), only the intensities were modified.

If we compare the experimental and computational spectra, we can propose a geometry for the reaction intermediary. The next spectra show us that the most similar structure compared with the experiment is the second case (from top to bottom), which is the square planar pyramid complex involving the Cas III-ia interacting with the cysteine without proton atom.

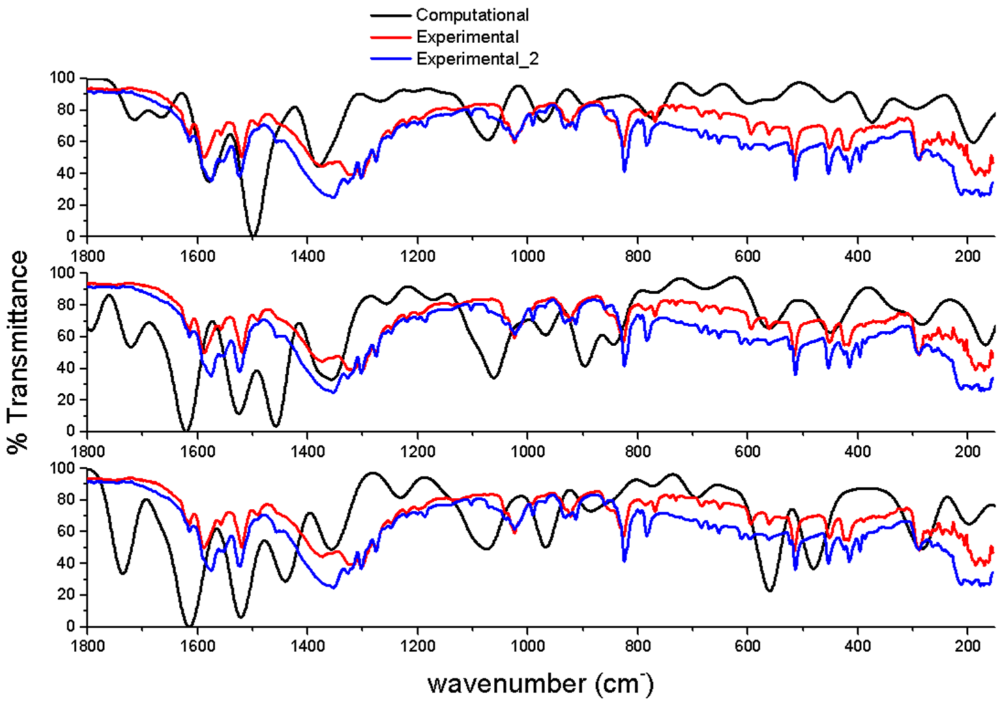

Figure S17. Comparison between computational and experimental IR spectra.

Signal Atoms	Type	Computational	Experimental
**Cu-H2O**	rocking	67.49	
**Cu-H2O**	asymmetric stretching	106.84	
**Cu-H2O**	symmetric stretching	135.63	
**O-Cu-O**	twisting	157.69	
**Cu-S**	symmetric stretching	178.75	175-188
**O-Cu-O**	wagging	201.84	
**Cu-S**	rocking	263.79	267-279
**N-Cu-N**	wagging	278.56	
**N-Cu-N**	twisting	286.99	
**N-Cu-N**	symmetric stretching	312.36	
**N-Cu-N**	scissoring	354.48	
**O-Cu-O**	asymmetric stretching	414.62	
**O-Cu-O**	symmetric stretching	452.94	
**C-S**	symmetric stretching	628.08	636-652
**C-S**	asymmetric stretching	702.07	692-707

Table S7. Values for octahedral geometry.

Signal Atoms	Type	Computational	Experimental
**Cu-S**	symmetric stretching	193.99	175-188
**Cu-Oax**	symmetric stretching	231.91	
**O-Cu-S**	asymmetric stretching	252.01	267-279
**N-Cu-N**	twisting	283.49	

**O-Cu-Oax**	twisting	311.84	
**Cu-S**	symmetric stretching	332.14	
**N-Cu-N**	scissoring	355.72	
**O-Cu-Oax**	asymmetric stretching	417.85	
**O-Cu-Oax**	symmetric stretching	430.56	
**N-Cu-N**	twisting	475.76	
**N-Cu-N**	asymmetric stretching	499.46	
**O-Cu-Oax**	rocking	544.66	
**N-Cu-N**	wagging	550.19	
**N-Cu-N**	asymmetric stretching	568.17	
**N-Cu-N**	symmetric stretching	575.19	
**O-Cu-Oax**	twisting	580.69	593-599
**C-S**	symmetric stretching	669.8	636-652

Table S8. Values for square planar pyramid geometry.

Electron Paramagnetic Resonance

We use the minimized structures for the EPR parameters calculation with the Orca 4.0.1 software. Calculations were performed at B3LYP/def2-SVP theory level. Table S9 shows the experimental and calculated values for the Cas III-ia and the CasIII-ia:Cysteine 1:1 system, testing the octahedral and square planar pyramid geometries.

Geometry	CasIII-ia	CasIII-ia:Cysteine 1:1	
		Octahedral	Square planar pyramid
**$g_{xx} = g_{yy}$**	2.0466		
**g_{zz}**	2.1458		
**$g_{xx} = g_{yy} = g_{zz}$**		2.1010	2.0755
**$A_{xx} = A_{yy}$ [MHz]**	105.26		
**A_{zz} [MHz]**	-185.99		
**$A_{xx}=A_{yy}=A_{zz}$ [MHz]**		104.57	23.85

Table S9. Calculated EPR parameters for CasIII-ia and the reaction CasIII-ia:Cysteine.

3. *Electron Transfer*

We use the fully optimized geometries at M05-2X/LanL2DZ theory level, then we used the Quantum Theory of Atoms In Molecules, taking the set of molecular orbitals of each molecule to compute the atomic properties of the electron density with the AIMAll software.

Figure S18 shows the spin density population of copper and sulfur atoms that describes the sulfhydryl radical formation. First, we studied the spin density change in four stages: 1) the Cas, 2) the octahedral complex formed when a cysteine molecule coordinates in the free axial position of Cas with a Cu-S bond length of 2.68 Å, 3) the intermediary square planar pyramid complex with a Cu-S bond length of 2.37 Å, and 4) when the cysteine gets far away from the

complex at 5 Å, and we observe the tetrahedral geometry of the Cas. This stage geometries and its spin density values for the copper atom (in blue) and the sulfur atom (in orange) are represented in Figure S18. In the case of copper, we observe that at the beginning has a value of 0.7 electrons, this value decreases when the reaction proceeds until the 4 stage in which it completely loses. In the opposite side, the sulfur atom does not have a spin density at the beginning, but its value increases when the reaction happens until it has a value of 0.9 electrons. It shows that copper has an excess of α spin density when sulfur atom approaches to the copper, the sulfur atom starts to lose β spin density that shares with the copper until it gets the change of oxidation state, then, the sulfur gets an excess of α spin density giving a sulfhydryl radical.

1. Cas III-ia

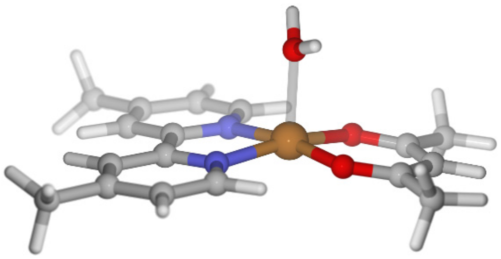

2. Octahedral complex

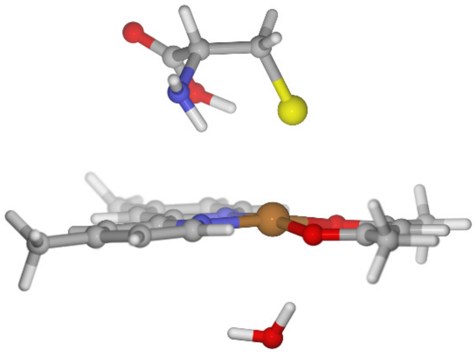

Cu-S distance = 2.68 Å

3. Square planar pyramid

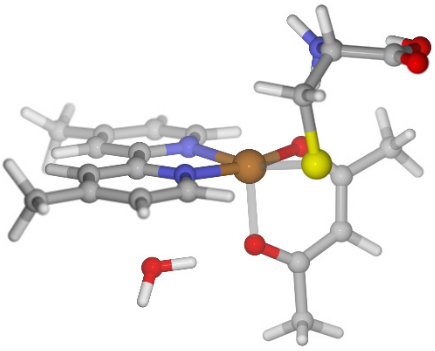

Cu-S distance = 2.37 Å

4. Tetrahedral complex + tyil radical

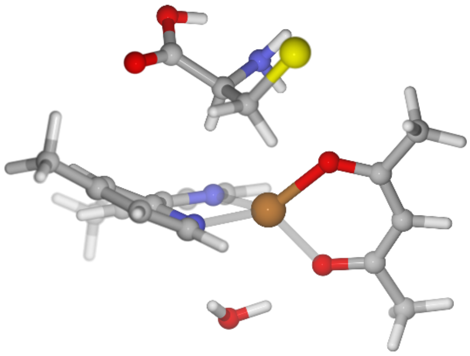

Cu-S distance = 5.0 Å

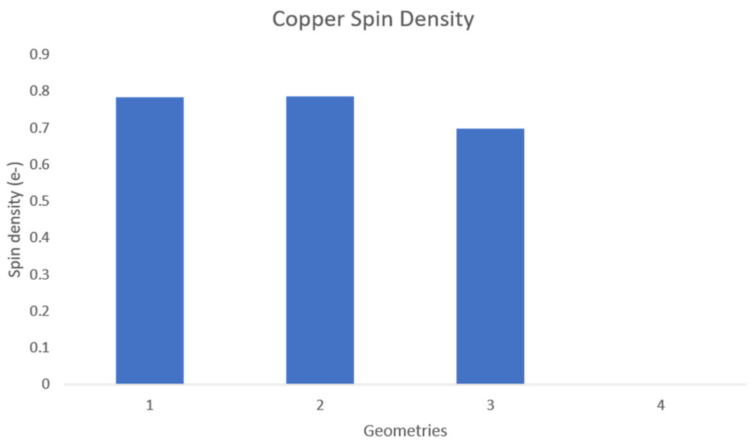

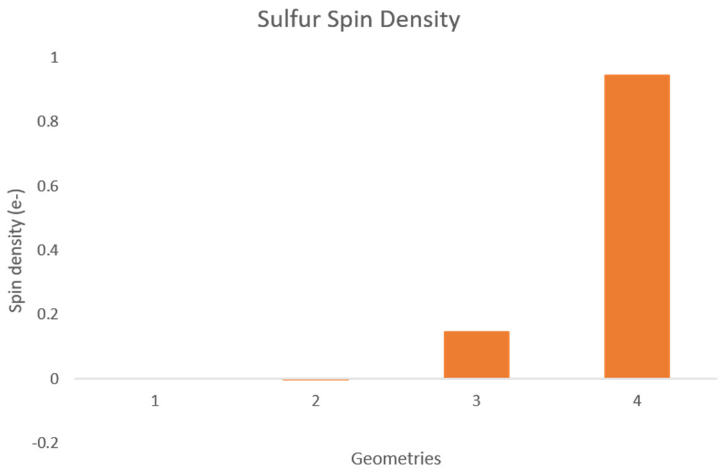

Figure S18. Spin density changes.

Then, we decided to study the process between stages 3 and 4 by scanning the change in spin density when the Cu-S bond length is changing too, the result is shown at the bottom of Figure S19. We noticed that there is not a gradual change in spin density, instead of that, the electron jumps instantaneously from sulfur atom to copper atom. This jump happens when there is a 3.95 Å bond length between the copper atom and the sulfur atom, this distance varies until almost 0.5 Å according to the substituents and the secondary ligand on the copper complex.

	Spin Density (u.a.)	
Cu-S distance (Å)	Cu	S
Cas III-ia	0.782737	-
2.68	0.78669	-0.002683
2.37	0.697838	0.145241
3	0.674146	0.185521
3.5	0.784289	-0.001474
3.6	0.782192	-0.001251
3.7	0.780671	-0.001052

3.8	0.779553	-0.000832
3.9	0.79617	-0.000184
4	0.00137	0.920494
4.5	0.00287	0.928122
5	0.001442	0.943536

Table S10. Spin density values for Cu and S atoms in the distance scan.

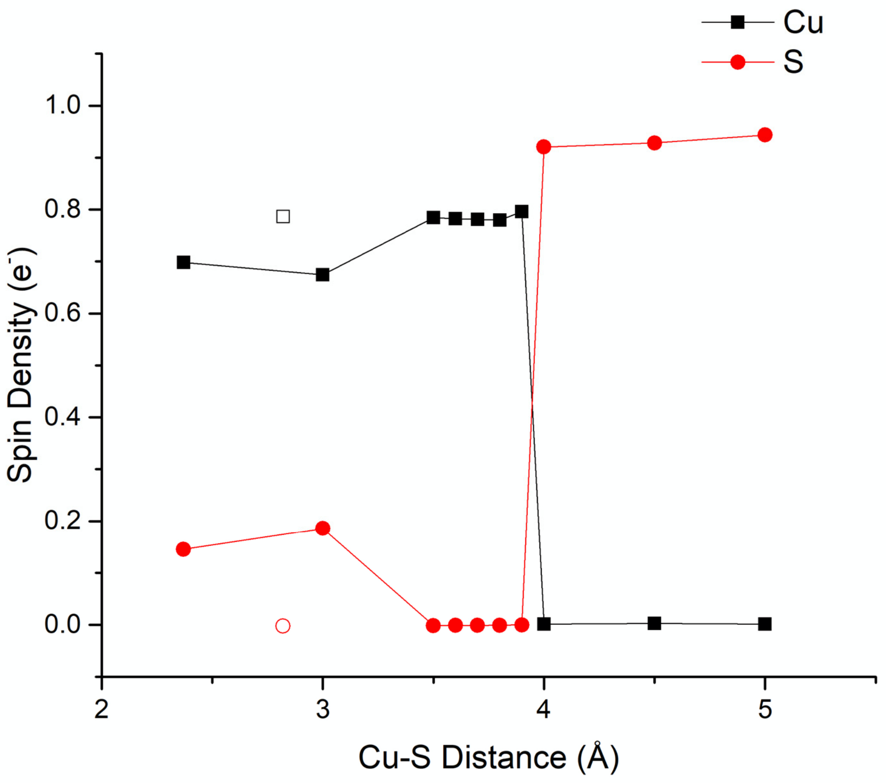

Figure S19. Spin density changes in Cu-S bond distance scan. Values in black for copper atom, and values in red for sulfur atom. Unfilled marks for octahedral geometry, for comparison.

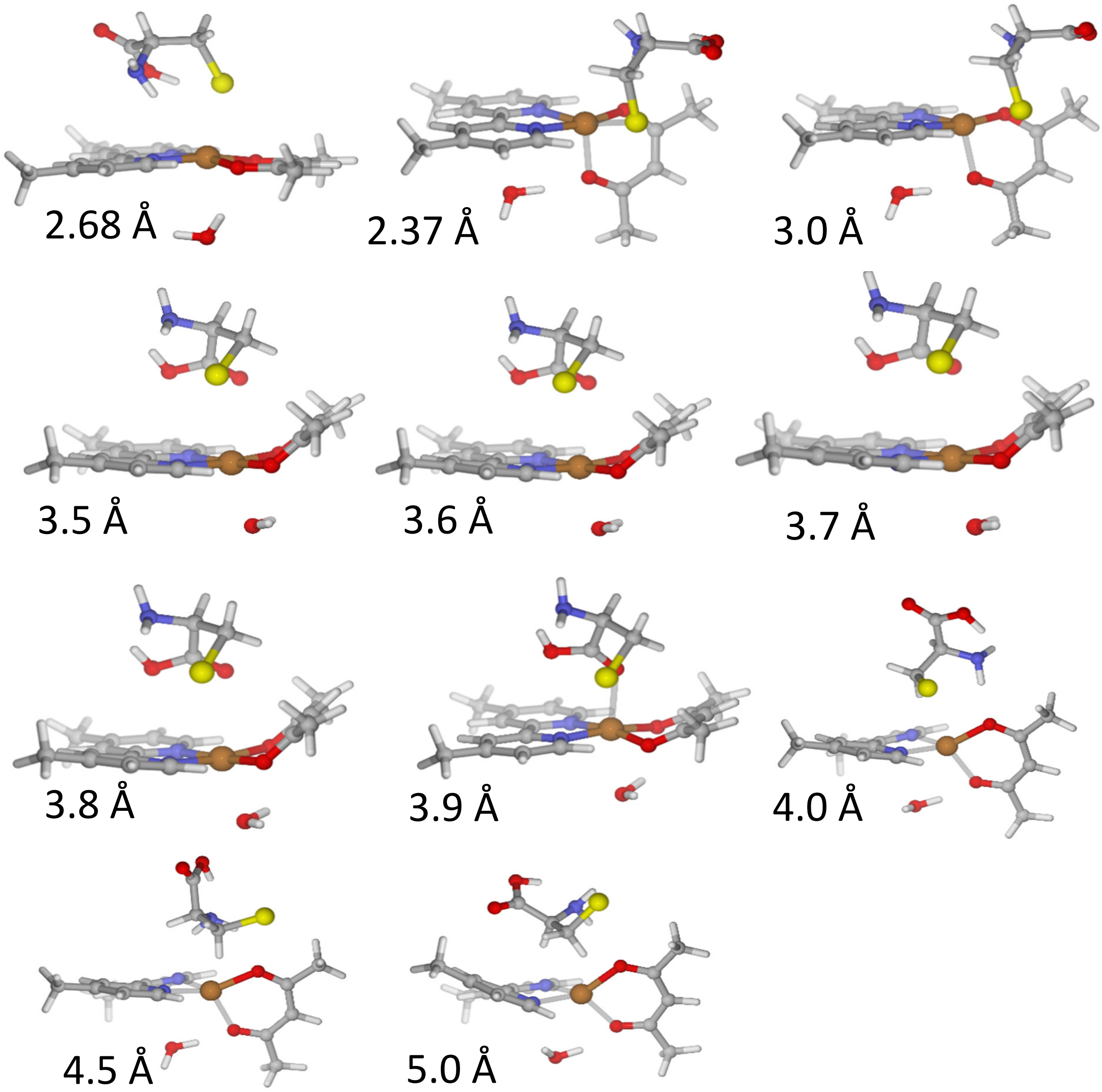

Figure S20. Geometries in the Cu-S bond distance scan.

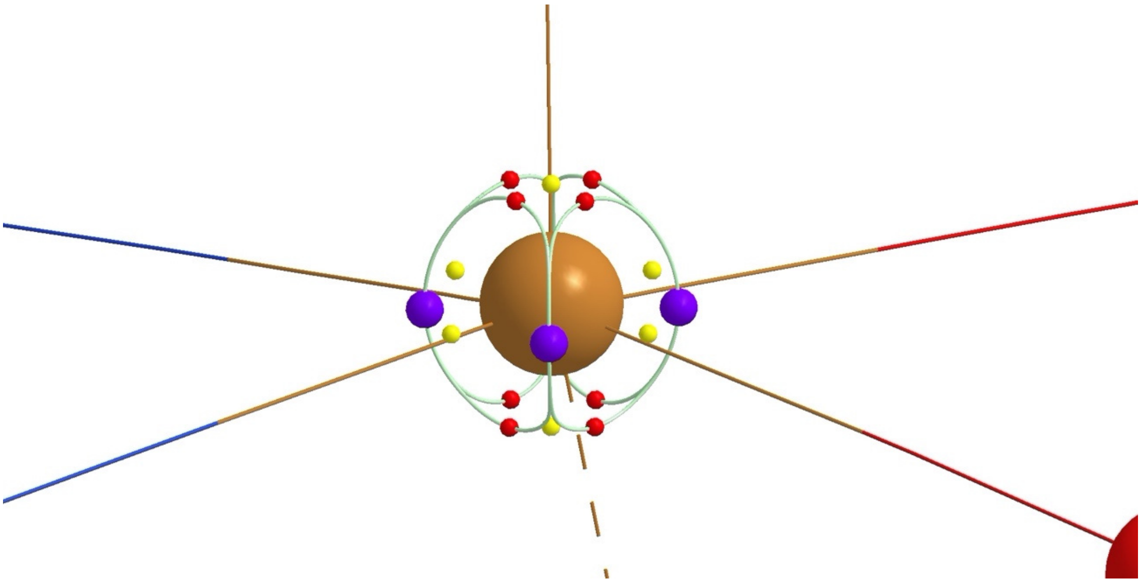

Figure S21. Atomic graph for geometry with Cu-S bond distance of 3.9 Å. (3, +3) CPs in purple, (3, +1) CPs in red and (3, −1) CPs in yellow.

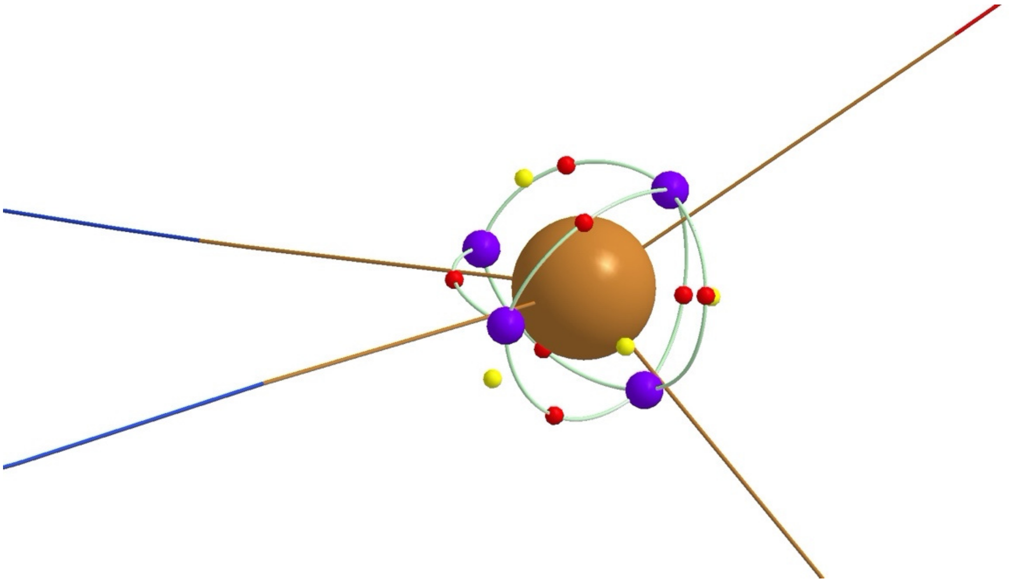

Figure S22. Atomic graph for geometry with Cu-S bond distance of 4.0 Å. (3, +3) CPs in purple, (3, +1) CPs in red and (3, −1) CPs in yellow.

References

1. Stoll, S.; Schweiger, A. EasySpin, a comprehensive software package for spectral simulation and analysis in EPR. *J. Magn. Reson.* **2006**, *178*, 42–55, doi:10.1016/j.jmr.2005.08.013.

2. Frisch, M.J.; Trucks, G.W.; Schlegel, H.B.; Scuseria, G.E.; Robb, M.A.; Cheeseman, J.R.; Scalmani, G.; Barone, V.; Mennucci, B.; Petersson, G.A.; et al. Gaussian 09 2009.

3. Bader, R. *Atoms in Molecules. A Quantum Theory.*; 1st ed.; Oxford University Press: New York, 1990;

4. Keith, T.A. AIMAll (Version 16.01.09), TK Gristmill Software, Overland Park, KS, USA, 2016, aim.tkgristmill.com.

5. Neese, F. The ORCA program system. *Wiley Interdiscip. Rev. Comput. Mol. Sci.* **2012**, *2*, 73–78, doi:10.1002/wcms.81.
